# A novel ER–microtubule-binding protein, ERLIN2, stabilizes Cyclin B1 and regulates cell cycle progression

**DOI:** 10.1038/celldisc.2015.24

**Published:** 2015-09-08

**Authors:** Xuebao Zhang, Juan Cai, Ze Zheng, Lisa Polin, Zhenghong Lin, Aditya Dandekar, Li Li, Fei Sun, Russell L Finley, Deyu Fang, Zeng-Quan Yang, Kezhong Zhang

**Affiliations:** 1 Center for Molecular Medicine and Genetics, Wayne State University School of Medicine, Detroit, MI, USA; 2 Karmanos Cancer Institute, Wayne State University School of Medicine, Detroit, MI, USA; 3 Department of Oncology, Wayne State University School of Medicine, Detroit, MI, USA; 4 Department of Pathology, Northwestern University Feinberg School of Medicine, Chicago, IL, USA; 5 Department of Immunology and Microbiology, Wayne State University School of Medicine, Detroit, MI, USA; 6 Department of Internal Medicine, Wayne State University School of Medicine, Detroit, MI, USA; 7 Department of Physiology, Wayne State University School of Medicine, Chicago, IL, USA; 8 Department of Biochemistry and Molecular Biology, Wayne State University School of Medicine, Detroit, MI, USA; 9 Department of Surgery, Northwestern University Feinberg School of Medicine, Chicago, IL, USA

**Keywords:** breast cancer, Cyclin B1, endoplasmic reticulum, ER lipid raft protein, microtubule, mitosis

## Abstract

The gene encoding endoplasmic reticulum (ER) lipid raft-associated protein 2 (ERLIN2) is amplified in human breast cancers. *ERLIN2* gene mutations were also found to be associated with human childhood progressive motor neuron diseases. Yet, an understanding of the physiological function and mechanism for ERLIN2 remains elusive. In this study, we reveal that ERLIN2 is a spatially and temporally regulated ER–microtubule-binding protein that has an important role in cell cycle progression by interacting with and stabilizing the mitosis-promoting factors. Whereas ERLIN2 is highly expressed in aggressive human breast cancers, during normal development ERLIN2 is expressed at the postnatal stage and becomes undetectable in adulthood. ERLIN2 interacts with the microtubule component α-tubulin, and this interaction is maximal during the cell cycle G2/M phase where ERLIN2 simultaneously interacts with the mitosis-promoting complex Cyclin B1/Cdk1. ERLIN2 facilitates K63-linked ubiquitination and stabilization of Cyclin B1 protein in G2/M phase. Downregulation of ERLIN2 results in cell cycle arrest, represses breast cancer proliferation and malignancy and increases sensitivity of breast cancer cells to anticancer drugs. In summary, our study revealed a novel ER–microtubule-binding protein, ERLIN2, which interacts with and stabilizes mitosis-promoting factors to regulate cell cycle progression associated with human breast cancer malignancy.

## Introduction

Endoplasmic reticulum lipid raft-associated protein 2 (ERLIN2), also known as SPFH2 or C8ORF2, is an endoplasmic reticulum (ER) membrane protein containing an evolutionarily conserved stomatin/prohibitin/flotillin/HflK/C (SPFH) domain. The *ERLIN2* gene resides on chromosome 8p11.2, a region that is frequently found altered in human breast cancer and several childhood onset autosomal recessive motor neuron diseases [1–4]. We and others have identified the *ERLIN2* gene as one of several candidate oncogenes within the 8p11-12 amplicon based on statistical analysis of copy number increase and over expression [1, 5–9]. Recent studies indicated that ERLIN2 may serve as a mediator of ER-associated protein degradation (ERAD). By binding to ERAD substrates such as the activated inositol trisphosphate receptors (IP3Rs), ERLIN2 mediates polyubiquitination and subsequent degradation of IP3Rs or 3-hydroxy-3-methylglutaryl-CoA reductase [10–12]. Our studies with human breast cancer cells suggest that ERLIN2 does not function as a mediator of ERAD although it interacts with the ER-resident insulin-induced gene 1 protein (INSIG1) to regulate activation of sterol regulatory element-binding protein (SREBP) 1c, the key regulator of *de novo* lipogenesis [13]. Through this regulation, ERLIN2 may help breast cancer cells maintain high levels of cytosolic lipid content and gain growth advantage under oncogenic stress conditions. A recent study from another group also demonstrated the interaction between ERLIN2 and SREBP–SCAP–INSIG1 complex [14]. However, this interaction was suggested to negatively regulate SREBP activation under conditions of cholesterol sufficiency. More recently, human genetic studies identified the *ERLIN2* gene mutations are associated with childhood neuronal diseases characterized by progressive weakness and spasticity of the lower extremities and intellectual disability [2–4]. Loss of ERLIN2 function by a splice-junction mutation of an *ERLIN2* transcript and the subsequent nonsense-mediated decay of *ERLIN2* mRNA causes a juvenile primary lateral sclerosis, a rare upper motor neuron disease [15, 16]. Although these studies have indicated that ERLIN2 is critically involved in both tumor development and childhood motor neuron degeneration, the role and mechanism of ERLIN2 in pathophysiology remain poorly understood.

Microtubules are components of the cytoskeleton that are essential in the regulation of cell division, cell motility, cell morphology and polarity as well as the intracellular distribution of organelles [17]. Microtubule stability is regulated in part by microtubule-associated proteins (MAPs), a heterogeneous family of proteins that bind to tubulin subunits of microtubules. Aberrant expression of MAPs, such as Tau, MAP2 and MAP4, is associated with the resistance phenotype of microtubule-targeting chemotherapy in various tumors, such as breast cancer, oral squamous cell carcinoma and malignant melanomas [18]. Related to the functions of microtubules, the G2/M-specific Cyclin B1 interacts with MAPs to regulate cell cycle progression [19, 20]. Cyclin B1 facilitates the transition of the cells from G2 to M phase but becomes unregulated in cancer cells where overexpression of Cyclin B1 may contribute to uncontrolled cell proliferation [21]. High expression of Cyclin B1 is associated with high breast tumor grade, larger tumor size and higher metastasis probability, and therefore, can be used as a tool to determine the prognosis of cancer patients [22, 23].

In this study, we demonstrated that ERLIN2 is a developmentally regulated, ER-localized MAP that interacts with Cyclin B1 complex during mitosis. ERLIN2 facilitates ubiquitination of Cyclin B1 at Lysine residue 63 (K63) and thus stabilizes Cyclin B1 in G2/M phase of cell cycle. Downregulation of ERLIN2 levels leads to G2/M phase arrest and represses human breast cancer cell proliferation and malignancy. Our study revealed the function and mechanism for a novel ER–microtubule-binding protein, ERLIN2, in regulating cell cycle progression and human breast cancer growth.

## Results

### ERLIN2 is a developmentally regulated protein

**Figure 1:**
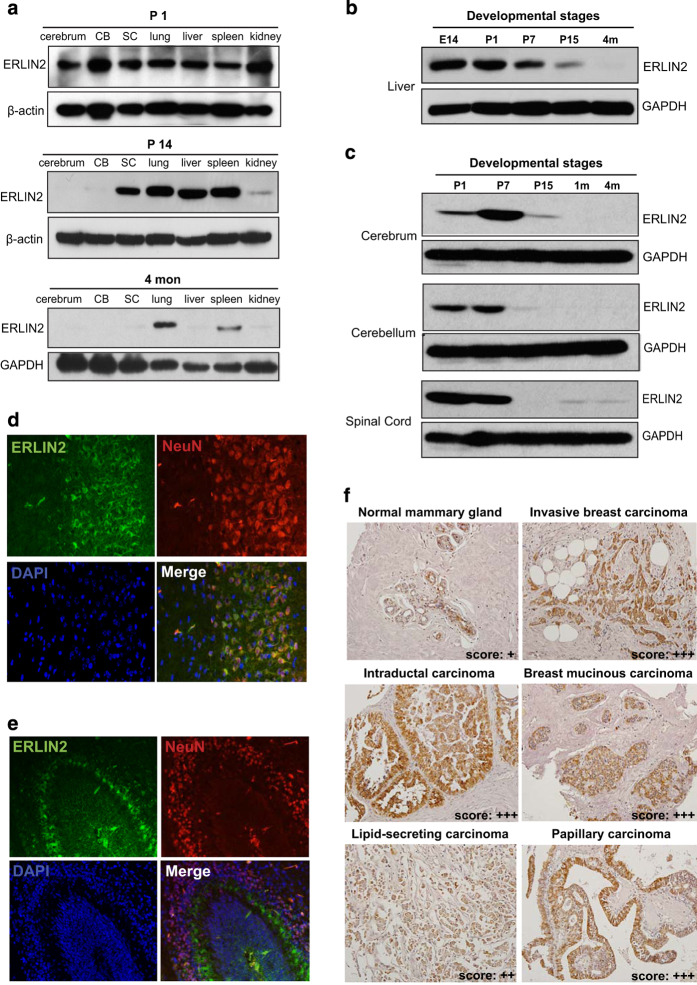
Expression of endoplasmic reticulum lipid raft-associated protein 2 (ERLIN2) is developmentally and spatially regulated. (**a**) Western blot analysis of ERLIN2 protein expression in cerebrum, cerebellum (CB), spinal cord (SC), lung, spleen, liver and kidney of mice at the ages ranging from postnatal day 1 (P1), P14, to 4 months (4 m). Levels of GAPDH or β-actin were determined as the loading controls. (**b**) Western blot analysis of ERLIN2 protein expression in the livers of mice at the ages ranging from embryonic day 14 (E14), P1, P7, P15, to 4 months (4 m). (**c**) Western blot analysis of ERLIN2 protein expression in cerebrum, CB, and SC of mouse brain tissues at the age ranging from P1, P7, P15, 1 m and 4 m. (**d**,**e**) IF staining of cortex neurons (**d**) and cerebellum (**e**) of brain tissue sections from mice at the age of P7. ERLIN2 was stained with green fluorescence, the neuronal marker NeuN was stained with red fluorescence, and nuclei were stained with DAPI. In cerebellum, positive ERLIN2 staining was only observed in Purkinje neurons but not in granule neurons. Magnification: ×200. (**f**) Immunohistochemical (IHC) staining of ERLIN2 in human breast cancer tissue array specimen obtained from US Biolab (http://www.usbiolab.com/). Representative images of IHC staining of normal human mammary gland tissues and different subtypes of aggressive human breast tumors are shown. ERLIN2 was stained as brown color (magnification: ×200). ERLIN2 staining were scored on a scale of 1–3: ERLIN2 staining in <50% of cells, +; intermediate staining in >50% of cells, ++; and strong staining in >50% of cells, +++.

Previously, we showed that the *ERLIN2* gene is highly expressed in a subset of aggressive breast cancer cell lines [13]. To test whether ERLIN2 expression is regulated spatially and temporally, we examined ERLIN2 expression profiles in a variety of mouse tissues including cerebrum, cerebellum, spinal cord, lung, spleen, liver and kidney under different developmental stages. ERLIN2 is highly expressed in cerebrum, cerebellum, spinal cord, lung, liver, spleen and kidney at postnatal day 1 (P1) (Figure 1a). From P14 to adult age, expression of ERLIN2 in cerebrum, cerebellum, spinal cord, liver and kidney was reduced to undetectable levels, suggesting that expression of ERLIN2 is developmentally regulated. To gain more insights into the developmentally regulated expression profile for ERLIN2, we examined expression of ERLIN2 in mouse fetal liver of embryonic stage day 14 (E14), postnatal liver of P1, P7, P15 and adult mouse liver of 4-month old. Expression of ERLIN2 in mouse liver reached a high level at the embryonic stage, gradually decreased with age, and was not detectable in the adult liver (Figure 1b). Because of the critical involvement of the *ERLIN2* gene mutations in progressive human childhood neuronal diseases [2, 3, 15, 24], we examined postnatal expression of ERLIN2 protein in mouse cerebrum, cerebellum and spinal cord from postnatal age P1 to the adult age of 4 months (Figure 1c). ERLIN2 expression reached peak levels in mouse cerebrum at P7, was significantly decreased by P15, and became undetectable by the age of 4 months. In cerebellum and spinal cord, ERLIN2 is moderately expressed at P1 and P7, and is undetectable after P15. We examined the cellular distribution of ERLIN2 in central nervous system (CNS) by immunofluorescent (IF) staining analysis. At P7 when ERLIN2 is highly expressed in brain tissue (Figure 1c), the distribution of ERLIN2 was found to overlap with neuronal marker NeuN positive neurons in cerebrum (Figure 1d). Interestingly, positive ERLIN2 staining was only observed in Purkinje neurons but not in granule neurons in cerebellum (Figure 1e). The specific expression and distribution of ERLIN2 in CNS neurons are consistent with the neurological clinical symptoms of human patients with ERLIN2 mutations [2, 3, 15, 24]. Moreover, human gene expression data available from the EMBL–EBI database suggests that expression of ERLIN2 is regulated by aging. Expression of the *ERLIN2* gene in human tissues of geriatrics and adults are significantly decreased, compared to those in juveniles, pediatrics and infants (Supplementary Figure S1), consistent with the temporally regulated *ERLIN2* gene expression profiles observed with the mouse model. In addition, we characterized ERLIN2 expression levels and distribution in different subtypes of human breast cancer tissues. Immunohistochemical staining of human breast cancer tissue array samples showed that ERLIN2 is highly expressed in aggressive breast cancer tumors, including invasive breast carcinoma, intraductal carcinoma, mucinous carcinoma, lipid secreting carcinoma and papillary carcinoma (Figure 1f). ERLIN2 protein signals are mainly distributed to the deranged invasive tumor tissues derived from mammary gland epithelial cells.

### ERLIN2 is associated with microtubules by binding to α-tubulin

**Figure 2:**
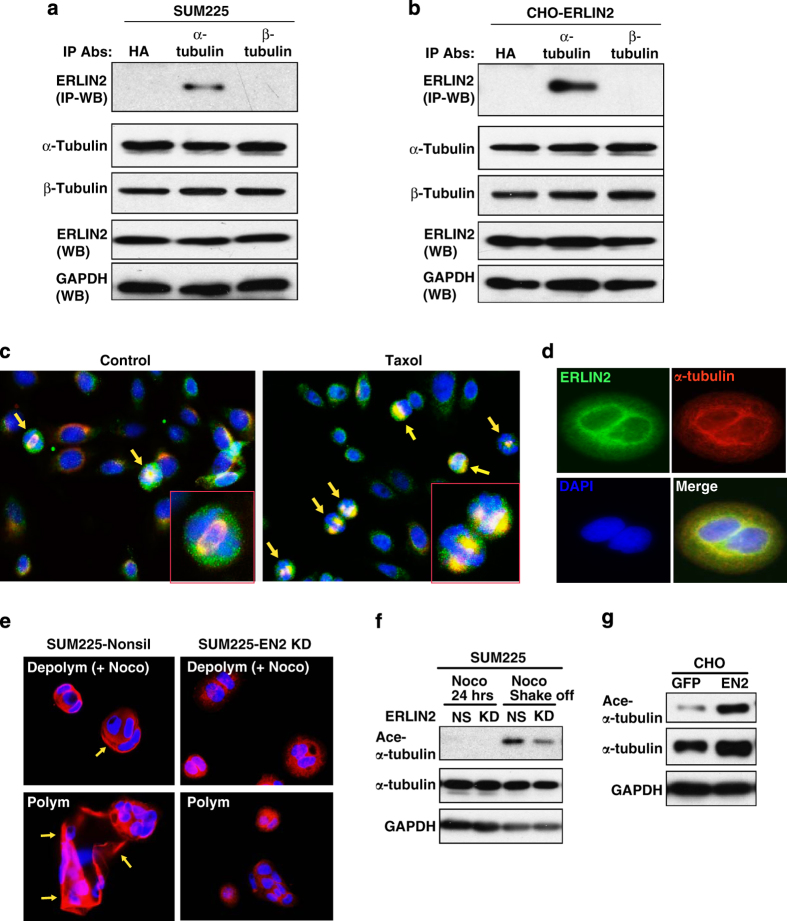
Endoplasmic reticulum lipid raft-associated protein 2 (ERLIN2) is associated with microtubules by interacting with α-tubulin. (**a**,**b**) Immunoprecipitation (IP)–western blot analysis of the interactions between ERLIN2, α-tubulin, or β-tubulin in SUM225 cells (A) or CHO cell expressing exogenous ERLIN2 (B). Total cell lysates from SUM225 or ERLIN2-expressing CHO cells were immunoprecipated with the anti-α-tubulin, anti-β-tubulin or anti-HA (negative control) antibody. The pull-down proteins were subjected to immunoblotting analysis using the anti-ERLIN2 antibody. The levels of ERLIN2 in total cell lysates were determined as the controls. (**c**) Immunofluorescent (IF) staining of ERLIN2 (green fluorescence) and acetylated α-tubulin (red fluorescence) in CHO cells treated with dimethyl sulfoxide (DMSO) or taxol (10 μm) for 1 h. Magnification: ×400. (**d**) IF staining of ERLIN2 (green fluorescence) and α-tubulin (red fluorescence) in SUM225 cells. Nuclei were stained with DAPI. Magnification: ×400. (**e**) Morphologic changes of microtubules in SUM225-nonsilence (nonsil) and ERLIN2-knockdown SUM225 (E2KD) cells. The microtubule morphology was shown by IF staining of α-tubulin (red fluorescence). The SUM225 cells were treated with 10 μm nocodazole for 2 h to depolymerize microtubules. After depolymerization, nocodazole was washed out to allow recovery of polymerization for 1 h. (**f**) Western blot analysis of α-acetylated-tubulin and total tubulin in nonsilence control SUM225 cells (nonsil) and ERLIN2-knockodown SUM225 cells (KD) treated with the vehicle DMSO or nocodazole (10 μm) for 24 h or treated with nocodazole for 24 h followed by removal of nocodazole for 15 min. GAPDH levels were determined as loading controls. (**g**) Western blot analysis of α-acetylated-tubulin and total tubulin in CHO cells overexpressing ERLIN2 (EN2) or GFP control. GAPDH levels were determined as loading controls. For **a**–**g**, the experiments were repeated at least three times, and representative data were presented.

Considering the developmental regulation of ERLIN2 and its association with breast cancer and neuronal diseases, we asked whether ERLIN2 is associated with microtubules or MAPs, the developmentally regulated cytoskeleton components that are critically involved in cancer cell proliferation and neuron development [25]. We first tested whether ERLIN2 can interact with α- and/or β-tubulin, the key components that make up microtubules [26], in the human breast cancer cell line SUM225 and in a stable CHO cell line overexpressing ERLIN2. Because microtubules can be depolymerized into α- and β-tubulins under the cold (0 °C) condition [27], immunoprecipitation (IP)–western blot analysis were performed with ice-cold protein lysates isolated from SUM225 or CHO cells to detect the interaction between ERLIN2 and tubulins. IP–western blot analysis indicated that α-tubulin, but not β-tubulin, interacts with ERLIN2 in SUM225 and ERLIN2-expressing CHO cells (Figure 2a and b). Further, we performed IF analysis to visualize the association of ERLIN2 with spindle microtubules in CHO cells. IF staining of ERLIN2 and acetylated-α-tubulin, a marker of polymerized microtubules [28], showed that ERLIN2 was partially colocalized with microtubules in the regions corresponding to the spindle poles or microtubule-organizing centers during mitosis (Figure 2c). Arrest of cells in mitosis by the microtubule stabilizer taxol resulted in increased ERLIN2 protein colocalized with spindle microtubules (Figure 2c), suggesting that ERLIN2 is a mitotic spindle microtubule-binding protein.

We wondered whether ERLIN2, a microtubule-binding protein, is involved in microtubule assembly or stability. IF analysis with SUM225, a human breast cancer cell line in which endogenous ERLIN2 is highly expressed [13], indicated that a majority of ERLIN2 protein was colocalized with α-tubulin in microtubules (Figure 2d). We then utilized an ERLIN2-knockdown SUM225 cell line to test the involvement of ERLIN2 in microtubule stability (Figure 2e, Supplementary Figure S2A). Upon challenge with the microtubule-depolymerizing agent nocodazole, most of the microtubules were disrupted in ERLIN2-knockdown cells, as reflected by a punctate cytoplasmic staining pattern of α-tubulin (Figure 2e, upper panel). In contrast, the microtubules in the nocodazole-treated control SUM225 cells remained intact, as thick bundle-like stabilized microtubules were present in the control cells. To test whether ERLIN2 can help polymerization of depolymerized microtubules, ERLIN2 knockdown and control cells were treated with nocodazole followed by removal of nocodazole for 1 h to allow recovery of microtubule polymerization. Whereas the control cells displayed a profound recovery of microtubule polymerization, as indicated by thick-bundle microtubule staining pattern, the ERLIN2-knockdown cells showed only partial recovery of polymerization (Figure 2e, lower panel), suggesting a role of ERLIN2 in promoting microtubule assembly and stabilization. To confirm the role of ERLIN2 in stabilizing microtubules, we examined levels of acetylated α-tubulin, a prominent marker of stabilized functional microtubules [28, 29], in ERLIN2-knockdown and control SUM225 cells upon exposing to or after release from nocodazole treatment and in ERLIN2-overexpressing and control CHO cells. Under the vehicle dimethyl sulfoxide treatment, the levels of acetylated α-tubulin in ERLIN2-knockdown SUM225 cells were comparable to those of SUM225 nonsilence control cells (Figure 2f). However, upon release from nocodazole treatment, levels of acetylated α-tubulin in SUM225-nonsilence cells were higher than those in the ERLIN2-knockdown cells, whereas the levels of total α-tubulin in both cell lines were comparable. These results implicated the role of ERLIN2 in stabilizing microtubules during the microtubule polymerization process. Furthermore, we determined the levels of acetylated α-tubulin in CHO cells overexpressing ERLIN2 or GFP control. ERLIN2-expressing CHO cells exhibited much higher levels of acetylated α-tubulin, compared with GFP-expressing CHO cells (Figure 2g), thus further confirming the role of ERLIN2 in stabilizing microtubules.

### ERLIN2 sediments with polymerized microtubules and interacts with α-tubulin via its SPFH domain

**Figure 3:**
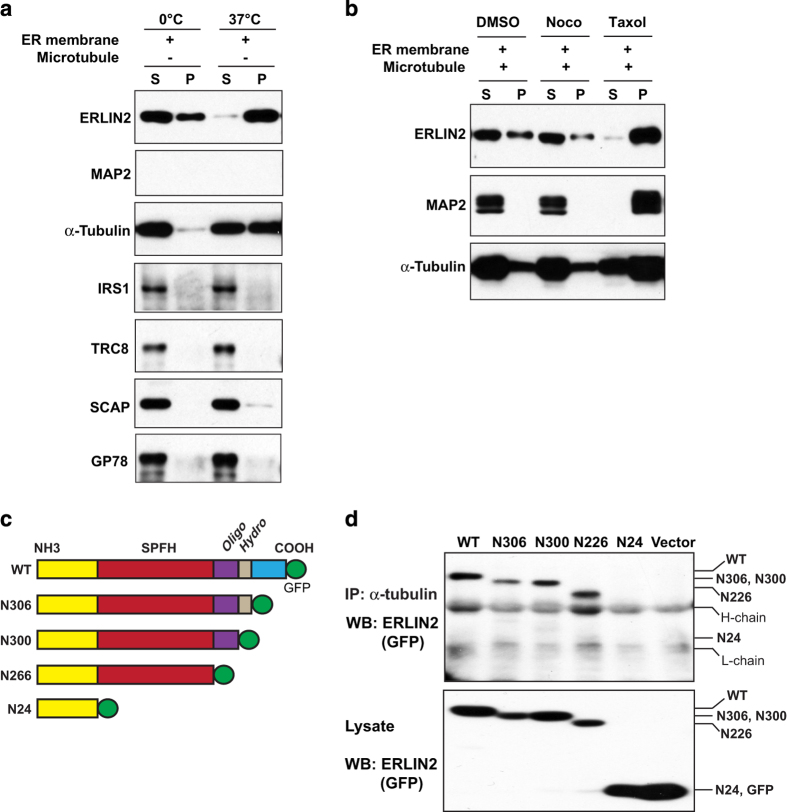
Endoplasmic reticulum lipid raft-associated protein 2 (ERLIN2) cosediments with microtubules and interacts with α-tubulin via its SPFH domain. (**a**,**b**) ERLIN2 co-assembles with microtubules *in vitro*. ERLIN2-enriched membrane proteins were mixed with mouse brain microtubule extracts at a ratio of 3:1 and then incubated at different conditions (0 °C, 37 °C, nocodazole or taxol treatment) to polymerize or depolymerize the microtubules. The samples were spun through a cushion buffer containing glycerol to collect polymerized microtubule pellets (P) and supernatants (S), which were subjected to immunoblotting analysis using the antibody against MAP2, ERLIN2 or α-tubulin. Levels of MAP2 were determined as a microtubule-associated protein control. (**c**) Schematic representation of different domains on ERLIN2 protein. SPFH, SPFH domain (22–226 amino acids (aa)); oligo, oligomerization domain (228–300aa); hydro, hydrophobic patch (301–306aa). Plasmid vectors were constructed to express ERLIN2 or its deletion mutations (n306, n300, n226, n24) fused with a C-terminal GFP. (**d**) Immunoprecipitation (IP)–Western blot analysis of the interaction between GFP-tagged ERLIN2 or various ERLIN2 deletion mutations and α-tubulin in CHO cells. Empty vector (pEGFP-N1 vector) was included as the control. Total cell lysates from transfected CHO cells were immunoprecipated with the anti-α-tubulin antibody. The pull-down proteins were subjected to immunoblotting analysis using anti-GFP antibody. The levels of GFP-tagged ERLIN2 variants in total cell lysates were determined as the controls. H-chain, IgG heavy chain; L-chain, IgG light chain. For **a**–**b** and **d**, the experiments were repeated at least three times, and representative data were presented.

To further characterize the interaction between ERLIN2 and microtubules, we performed microtubule cosedimentation assays using microtubules extracted from mouse brain tissues and ERLIN2-enriched membrane protein fraction extracted from ERLIN2-overexpressing cells. ERLIN2-enriched membrane protein fractions were incubated at 0 °C (on ice) to induce microtubule depolymerization or at 37 °C to induce microtubule polymerization as previously described [27, 30]. It is known that membrane protein extractions contain a certain level of tubulin monomers, which is presumably attributed to the physical association between ER membrane and microtubules [31]. In the absence of exogenous microtubule extracts, both ERLIN2 and endogenous α-tubulin were detected in the supernatants collected from the cold-incubated ERLIN2-enriched membrane protein fractions after the high-speed centrifugation (Figure 3a). When the membrane protein fractions containing endogenous tubulins were incubated at 37 °C to induce microtubule polymerization, both α-tubulin and ERLIN2 were present in the pellets (Figure 3a). These results were consistent with the observation that ERLIN2 is associated with α-tubulin during microtubule polymerization (Figure 2). As controls, the cytoplasmic protein IRS1 or other ER membrane proteins, including SCAP, TRC8 and GP78, remained in the supernatants separated from the polymerized membrane protein pellets, whereas the microtubule-associated protein MAP2 was not present in the membrane protein fractions in the absence of exogenous microtubule extracts (Figure 3a). To further delineate the association of ERLIN2 with microtubules, ERLIN2-enriched membrane protein extractions were incubated with exogenous microtubule extracts in the presence of the depolymerizing agent nocodazole or the microtubule stabilizer taxol. In the presence of nocodazole, the majority of ERLIN2 and α-tubulin were detected in the supernatants separated from the microtubule pellets (Figure 3b). When the microtubule and ER membrane fractions were stabilized with taxol, both ERLIN2 and α-tubulin were present in the microtubule pellets. As a MAP control, MAP2 was cosedimented with both monomerized and polymerized microtubule proteins in the supernatants or pellets. Taken together, these results suggest that ERLIN2 is associated with α-tubulin and polymerized microtubules.

The structure of ERLIN2 protein consists of a SPFH domain, an oligomerization domain, and a hydrophobic patch domain (Figure 3c) [2]. On the basis of the predicted ERLIN2 topology, the N-terminus of ERLIN2 is located in either the cytosol or ER lumen in two speculative models (Supplementary Figure S3). Two candidate membrane-spanning (transmembrane) segments are located at the amino acids 4 to 24 and the amino acids 38 to 58, respectively. In both ERLIN2 topological models, part of the SPFH domain is exposed to the cytosol (Supplementary Figure S3). To determine which domain is responsible for the interaction between ERLIN2 and microtubule α-tubulin, we constructed a series of expression vectors expressing GFP-tagged full-length ERLIN2 and its truncated mutants and expressed them in CHO cells (Figure 3d). IP–western blot analysis showed that full-length ERLIN2 and its truncated forms, including N306 (C-terminal domain deletion), N300 (hydrophobic patch deletion), and N226 (oligomerization domain deletion), exhibited binding activities to α-tubulin (Figure 3d). In contrast, the ERLIN2 truncated version N24, in which the SPFH domain was deleted, exhibited no binding activity to α-tubulin, suggesting that the SPFH domain is required for the interaction between ERLIN2 and α-tubulin.

### ERLIN2 complexes with Cyclin B1, Cdk1 and α-tubulin in the G2/M phase

**Figure 4:**
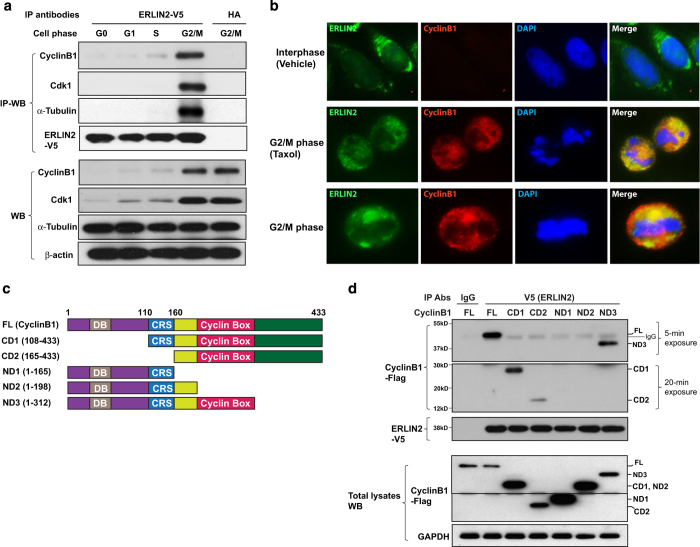
Endoplasmic reticulum lipid raft-associated protein 2 (ERLIN2) complexes with Cyclin B1, Cdk1 and α-tubulin in G2/M phase. (**a**) IP–western blot (IP–WB) analysis of the interactions between ERLIN2, Cyclin B1, Cdk1 or α-tubulin in ERLIN2 (tagged with V5)-expressing CHO cells synchronized at G0, G1, S or G2/M phase. Total cell lysates were immunoprecipated with the anti-V5 antibody or anti-HA (negative control). The immunoprecipated proteins were subjected to immunoblotting analysis using the anti-Cyclin B1, anti-Cdk1 or anti-α-tubulin antibody. The levels of Cyclin B1, Cdk1, α-tubulin and β-actin in total cell lysates were determined as the controls. (**b**) Immunofluorescent (IF) staining of ERLIN2 (green fluorescence) and Cyclin B1 (red fluorescence) in the CHO cell line stably expressing ERLIN2. The cells were treated with taxol (10 μm) or the vehicle dimethyl sulfoxide (DMSO) for 1 h. Nuclei were stained with DAPI (blue). The images on the right were for a representative CHO cell in G2/M phase stained for ERLIN2, Cyclin B1 and nuclei. Magnification: ×400. (**c**) Domain structures of full-length (FL) Cyclin B1 protein and its truncated mutants. Cyclin B1 contains an N-terminal destruction box (DB), followed by a cytoplasmic retention sequence (CRS) and a cyclin box domain. The amino acid number of each isoform was indicated. (**d**) IP–western blot analysis of the interactions between ERLIN2, Cyclin B1 (FL), and Cyclin B1 truncated isoforms (illustrated in **c**) in CHO cells. Plasmid vectors expressing flag-tagged Cyclin B1 and truncated Cyclin B1 isoforms were transfected into the CHO cell line stably expressing V5-tagged ERLIN2. Total cell lysates from transfected CHO cells were immunoprecipated with the anti-V5 antibody or rabbit IgG (negative control). The pull-down proteins were probed with the anti-flag antibody to detect the association of ERLIN2 with Cyclin B1 or its truncated forms. The pull-down proteins were probed with the anti-V5 antibody for the loading controls. Because of the different strengths of interactions between ERLIN2 and the Cyclin B1 truncated forms, both short (5 min)- and long (20 min)-time film exposure images were included to identify the interaction signals. The levels of Cyclin B1 or its truncated forms and GAPDH in total cell lysates were determined as the controls (lower panels).

Since ERLIN2 is associated with spindle microtubules (Figure 2), we asked whether ERLIN2 interacts with mitosis-regulatory components during cell cycle progression. We established an ERLIN2-expressing CHO stable cell line and synchronized the cells at G0, G1 or S phase through a serum starvation-release approach. Cells were synchronized at G2/M phase by the nocodazole treatment for 16 h. IP–western blot analysis indicated that ERLIN2 interacts with the key mitotic regulatory protein Cyclin B1 in the G2/M phase, but not the interphase (Figure 4a). Cyclin B1 is known to complex with Cdk1 to form M phase-promoting factor that is associated with spindle microtubules during the M phase [32, 33]. IP–western blot analysis showed that ERLIN2 specifically complexes with Cdk1 in the G2/M phase. Similar to the interaction dynamics of ERLIN2–Cyclin B1/Cdk1, ERLIN2 strongly interacts with α-tubulin in the G2/M phase (Figure 4a). These results suggest that ERLIN2 is associated with the Cyclin B1/Cdk1 complex during mitosis. To visualize the interaction between ERLIN2 and Cyclin B1, we performed IF staining of ERLIN2 and Cyclin B1 in the ERLIN2-expressing CHO stable cell line. During the interphase, no merged ERLIN2–Cyclin B1 signals were detected (Figure 4b). In contrast, in the G2/M phase, a significant fraction of ERLIN2 and Cyclin B1 are colocalized to the microtubule spindles (Figure 4b).

To further refine our understanding of the molecular interaction between ERLIN2 and Cyclin B1, we constructed truncated Cyclin B1 proteins in which the domains containing destruction box (DB), cytoplasmic retention sequence (CRS), or cyclin box are deleted (Figure 4c). IP–western blot analysis indicated that the Cyclin B1 truncated mutants with the cyclin box deleted were unable to complex with ERLIN2 protein (Figure 4d), suggesting that cyclin box mediates the interaction between ERLIN2 and Cyclin B1. Interestingly, truncated Cyclin B1 proteins containing the cyclin box but lacking DB or CRS domains exhibited reduced binding activities to ERLIN2, compared with full-length Cyclin B1 or the truncated form (ND3) containing DB, CRS and cyclin box, suggesting that the DB and CRS domains are required for optimal interaction between Cyclin B1 and ERLIN2.

ERLIN2 proteins across the mammalian species possess three conserved cyclin-binding motifs in the SPHF domain (Supplementary Figure S4) [34]. We examined whether ERLIN2 binds Cyclin B1 via the SPFH domain by IP–western blot analysis (Supplementary Figure S5). While full-length ERLIN2 and its truncated forms, including N306 (C-terminal domain deletion), N300 (hydrophobic patch deletion), and N226 (oligomerization domain deletion), exhibited binding activities to Cyclin B1, the ERLIN2 truncated version N24, in which the SPFH domain was deleted, exhibited no binding activity to Cyclin B1 (Supplementary Figure S5B), suggesting that the SPFH domain is required for the interaction between ERLIN2 and Cyclin B1.

### ERLIN2 facilitates K63-linked ubiquitination and stabilization of Cyclin B1 protein in G2/M phase

**Figure 5:**
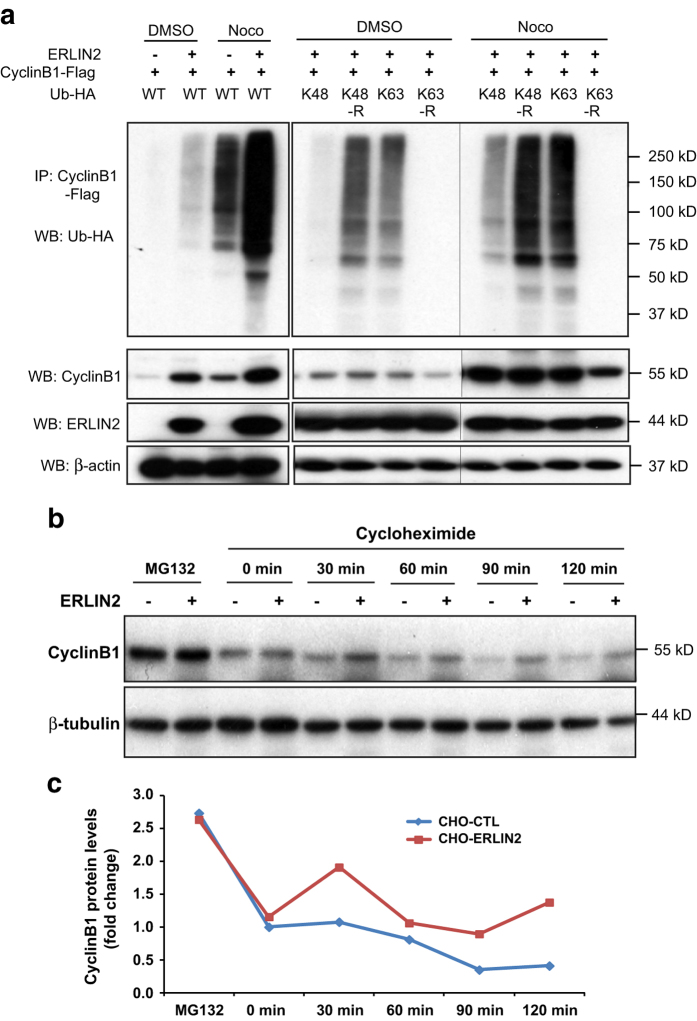
Endoplasmic reticulum lipid raft-associated protein 2 (ERLIN2) promotes K63-linked ubiquitination of Cyclin B1 and stabilizes Cyclin B1 in G2/M phase. (**a**) Immunoprecipitation (IP)–western and western blot (WB) analyses of Cyclin B1 ubiquitination and Cyclin B1 protein levels in ERLIN2-expressing CHO cells. Expression vectors for flag-tagged Cyclin B1 and HA-tagged ubiquitin (Ub) or its isoforms, including K48, K63, K48R and K63R, were cotransfected into the CHO cells stably expressing exogenous ERLIN2 or control CHO cells. The CHO cells were then treated with the vehicle dimethyl sulfoxide (DMSO) or Nocodazole to synchronize the cells in the G2/M phase. The cell lysates were immunoprecipated with the anti-flag antibody to pull down Cyclin B1 and then probed with the anti-HA antibody to detect ubiquitination. The lower panel showed the western blot analysis with the protein lysates from the same cells for the levels of Cyclin B1, ERLIN2 and β-actin proteins. (**b**) Cycloheximide translation inhibition analysis of Cyclin B1 protein stability. ERLIN2-overexpresing (CHO-13) and control CHO cell lines were treated with 10 μg ml^−1^ cycloheximide and collected at 0, 30, 60, 90 and 120 min after the treatment. Before addition of cycloheximide, cells were arrested in G2/M phase by treating with 120 ng ml^−1^of nocodazole for 16 h. To confirm that the decrease in Cyclin B1 levels is a result of proteasome-mediated protein degredation, 5 μm of MG132 were added together with nocodazole. The protein lysates collected from the CHO cells were subjected to western blot analysis to determine the levels of Cyclin B1 and β-actin (loading control). (**c**) Quantification of fold changes in Cycline B1 protein levels in ERLIN2-overexpresing and control CHO cells following cycloheximide treatment. The protein signal intensities, determined by western blot analysis as shown in **c**, were quantified using Quantity One software. After normlization to β-actin levels, fold changes of Cyclin B1 protein levels were calculated by comparing the Cyclin B1 protein levels to that in MG132-treated CHO control cells (defined as 1). For **a**–**c**, the experiments were repeated at least three times, and representative data were presented.

Next, we delineated the functional significance of the interaction between ERLIN2 and Cyclin B1/Cdk1 in the mitotic phase. During the M phase, Cyclin B1 levels first rise to allow the accumulation of Cyclin B1/Cdk1 complexes [35]. The mitotic spindle assembly checkpoint inhibits ubiquitin ligase activity of the anaphase-promoting complex/cyclosome (APC/C) and thereby preventing Cyclin B1 destruction and Cdk1 inactivation [36, 37]. The sustained high Cyclin B1/Cdk1 activity allows cells to stay in mitosis as long as is required for all chromosomes to attach to the mitotic spindle. Thereafter, progressive loss of Cyclin B1/Cdk1 activity through proteasome-mediated degradation is essential for the M phase exit and completion of cell division. To determine whether ERLIN2 is involved in Cyclin B1 degradation or stabilization during the M phase, we examined ubiquitination of Cyclin B1 in the ERLIN2-expressing CHO stable cell line. The CHO cells were treated with nocodazole to arrest most of the cells at the G2/M phase before being subjected to IP–western blot analysis to detect Cyclin B1 protein ubiquitination. Upon the nocodazole or vehicle (dimethyl sulfoxide) treatment, ERLIN2-expressing CHO cells exhibited significantly increased Cyclin B1 ubiquitination activity, compared with the control CHO cells (Figure 5a). Importantly, the levels of Cyclin B1 protein in the ERLIN2-expressing cells were significantly higher than those in the control cells expressing trace levels of endogenous ERLIN2 (Figure 5a, lower panel). These results suggest that ERLIN2 may promote Cyclin B1 ubiquitination and stabilization in the G2/M phase. To further delineate the role of ERLIN2 in Cyclin B1 ubiquitination and stabilization, we characterized the Ub chain of Cyclin B1 facilitated by ERLIN2. ERLIN2 and Cyclin B1 were co-expressed in CHO cells along with one of four different mutant isoforms of Ub: Ub carrying a single lysine (K) residue at position 48 (K48), Ub in which K48 was mutated to arginine (R) but all other lysine residues were intact (K48R), Ub carrying a single lysine residue at position 63 (K63), or Ub in which K63 was mutated to R but all other lysine residues were intact (K63R; Figure 5a). Upon nocodazole treatment, coexpression of K48 led to low levels of Cyclin B1 ubiquitination in ERLIN2-expressing CHO cells, while expression of K48R caused much higher levels of Cyclin B1 ubiquitination. In contrast, expression of K63 led to markedly increased Cyclin B1 ubiquitination in ERLIN2-expressing CHO cells, compared with expression of K48. However, when K63R was expressed, Cyclin B1 ubiquitination in ERLIN2-overexpressing cells was diminished (Figure 5a), indicating that ERLIN2 facilitates Cyclin B1 K63‐linked, but not K48‐linked, polyubiquitination. It is known that cellular proteins conjugated to K48‐linked Ub chains are targeted to proteasomes for degradation, whereas proteins conjugated to K63‐ubiquitin chains are protected from proteasome-mediated degradation [38, 39]. Consistent with this notion, overexpression of ERLIN2 increased levels of Cyclin B1 protein, likely due to the K63-linked ubiquitination of Cyclin B1 (Figure 5a, lower panel). Moreover, coexpression of K48 did not decrease Cyclin B1 protein levels in the ERLIN2-expressing cells. In correlation with diminished Cyclin B1 ubiquitination, levels of Cyclin B1 protein were decreased in CHO cells expressing K63R Ub.

To confirm the role of ERLIN2 in stabilizing Cyclin B1 protein, we monitored Cyclin B1 protein abundance in ERLIN2-expressing and control CHO cell lines following cycloheximide-mediated translation inhibition. ERLIN2-overexpressing and control CHO cells were arrested in G2/M phase by nocodazole treatment before they were treated with cycloheximide. Although Cyclin B1 protein in the control CHO cells exhibited a time-dependent degradation pattern, degradation of Cyclin B1 protein in ERLIN2-overexpressing CHO cells was inhibited following cycloheximide treatment (Figure 5b and c). As a control, degradation of Cyclin B1 protein following cycloheximide treatment was prevented by addition of the proteasome inhibitor MG132, suggesting that stabilization of Cyclin B1 protein in ERLIN2-overexpressing cells may result from inhibition of proteasome-mediated protein degredation. Taken together, these results implicate that ERLIN2 stabilizes Cyclin B1 protein in G2/M phase and this stabilization is facilitated through K63-linked ubiquitination by ERLIN2.

**Figure 6:**
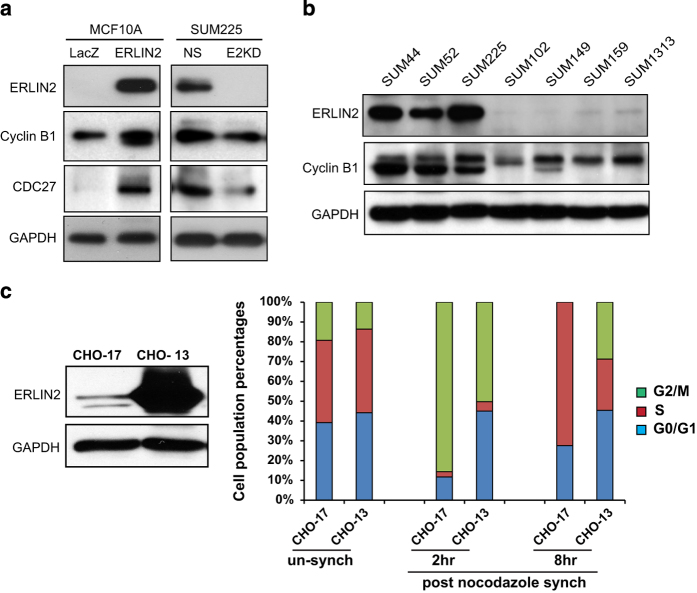
Functional implications of endoplasmic reticulum lipid raft-associated protein 2 (ERLIN2) in human breast cancer cells and CHO cells. (**a**) Western blot analysis of ERLIN2, Cyclin B1, Cdc27 and GAPDH in ERLIN2-overexpressing MCF10A cells, the LacZ-expressing control MCF10A cells, ERLIN2-knockdown SUM225 cells (E2KD) and nonsilence control SUM225 cells (NS). (**b**) Western blot analysis of ERLIN2, Cyclin B1 and GAPDH protein levels in human breast cancer cell lines. (**c**) Flow cytometry analysis (FACS) of cell cycle profiles in ERLIN2-overexpressing and control CHO cells. Two CHO cell lines, CHO-17 and CHO-13, which stably express different levels of ERLIN2, were established using a lentiviral-mediated expression system. Levels of ERLIN2 protein in CHO-17 and CHO-13 were determined by western blot analysis. CHO-17 and CHO-13 cells were treated with nocodazole for 16 h to arrest most cells at prometaphase by depolymerizing spindle microtubules. The culture medium containing nocodazole was then washed out and replaced with normal culture medium to allow recovery of functional spindle microtubules at different time points. Flow cytometry analysis (FACS) analysis was conducted with the cells collected at 2 and 8 h after nocodazole removal to profile the percentages of cells in G0/G1, S and G2/M phases upon recovery from nocodazole treatment. For **a–c**, the experiments were repeated at least three times and representative data were presented.

Next, we evaluated levels of phosphorylated Cdc27 (APC3), a direct target of Cyclin B1/Cdk1 during M phase [40], in the ERLIN2-expressing CHO cells. Supporting the role of ERLIN2 in stabilizing Cyclin B1/Cdk1 through K63-linked ubiquitination, coexpression of K63 or K48R led to much higher levels of phosphorylated Cdc27 proteins in ERLIN2-expressing CHO cells than coexpression of K63R or K48 (Supplementary Figure S6). Supporting the roles of ERLIN2 in stabilizing Cyclin B1 protein and enhancing Cyclin B1/Cdk1 function in breast cancer cells, levels of Cyclin B1 and phosphorylated Cdc27 were increased in ERLIN2-expressing MCF10A cells but decreased in ERLIN2-knockdown SUM225 cells, compared with their controls (Figure 6a). Further, correlating with the high expression levels of ERLIN2, Cyclin B1 protein levels in human breast cancer cell lines, SUM44, SUM52 and SUM225, were markedly higher than those in the breast cancer cell lines with low ERLIN2 levels ​(Figure 6b). As ERLIN2 stabilizes Cyclin B1/Cdk1 complex in the G2/M phase, we wondered whether ERLIN2 overexpression can prolong the M phase during cell cycle progression. To test this possibility, we profiled cell division cycles of the CHO cells expressing high levels of exogenous ERLIN2 (Figure 6c). The ERLIN2-overexpressing CHO cell line was treated with nocodazole for 16 h to destabilize spindle microtubule complexes, which can lead to most of the cells arrested in prometaphase [41]. Nocodazole was then removed to allow mitotic cells to progress synchronously into the next cell cycle. FACS analysis indicated that ~50% of the CHO cells overexpressing ERLIN2 (CHO-13) were arrested in the G2/M phase at 2 h after nocodazole removal, whereas over 85% of the CHO cells expressing low levels of ERLIN2 (CHO-17) were arrested in G2/M phase (Figure 6c, Supplementary Figure S7). The resistance to nocodazole-induced spindle microtubule destabilization and cell cycle synchronization, as exhibited in the CHO-13 cells, may reflect the role of ERLIN2, as a MAP protein, in stabilizing microtubules. Furthermore, at 8 h after nocodazole removal, all of CHO-17 cells were released from the G2/M phase arrest. However, over 28% of CHO-13 cells still remained in the G2/M phase. The persistent G2/M phase of CHO-13 is likely owing to sustained high levels of Cyclin B1/Cdk1 protein complex stabilized by ERLIN2 via K63-linked ubiquitination of Cyclin B1.

### Downregulation of ERLIN2 represses human breast cancer cell proliferation and malignancy

**Figure 7:**
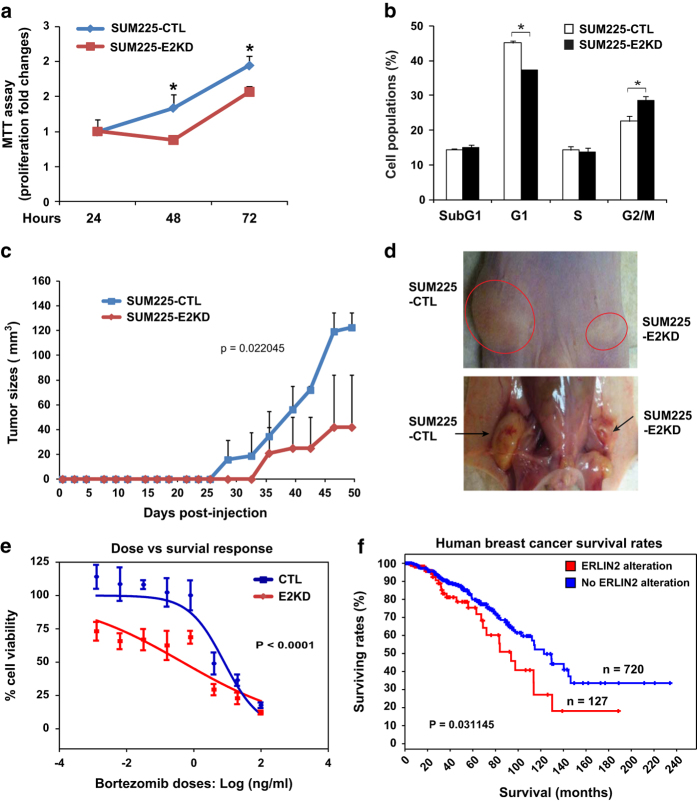
Knockdown of endoplasmic reticulum lipid raft-associated protein 2 (ERLIN2) represses human breast cancer cell proliferation and malignancy *in vivo*. (**a**) Cell proliferation assay (MTT) of ERLIN2-knockdown (E2KD) and control (CTL) SUM225 cells. The same numbers of ERLIN2-knockdown and nonsilence control SUM225 (~5 000 cells per well) were seeded. The assay was performed with the cells after 24, 48, and 72 h of culture. The error bars denote the standard deviation (*n*=4 biological repeats). (**b**) Cell cycle flow cytometry analysis (FACS) assay of ERLIN2-knockdown and control SUM225 cells after 6 days of culture. VybrantDyeCycle Orange Stain was used to stain live cell DNA content. The trypsinized cells were stained and subjected to a fluorescence-activated cell sorter to quantify cell populations in different cell cycle phases. (**c**) Growth rates of ERLIN2-knockdown and control human breast cancer tumors in the xenograft nude mice. The same numbers of ERLIN2-knockdown and control human breast cancer SUM225 cells were orthotopically transplanted into the mammary fat pads of the left and right flanks of nude mice. Tumor growth (sizes) were monitored every 3 days. Mice were euthanized at 7 weeks after injection. (**d**) Appearance of ERLIN2-knockdown and control breast tumors developed in the nude mice at 7 weeks after the implantation. (**e**) SUM225 cell dose–response to Bortezomib treatment. ERLIN2-knockdown and control SUM225 cells were treated with Bortezomib at the concentrration ranging from 0.00128, 0.0064, 0.032, 0.16, 0.8, 4, 20, to 100 ng ml^−1^ (serial 5:1 dilutions) for 72 h. After the incubation period, MTT assay was performed to determine cell viability. Each treatment point was performed in quadruplicate and the cell survival dose–response curves were generated through nonlinear regression based on log(dose) vs cell viability response (variable slope). The IC_50_ (inhibitory concentration of Bortezomib with 50% cell viability) values are 8.027 for the control cells and 0.4675 for the ERLIN2-knockdown cells. Two-way analysis of variance (ANOVA) grouped analysis *P*<0.0001. (**f**) Survival rates of human breast cancer patients with or without ERLIN2 gene alteration. In 959 all complete primary breast cancer cases provided by TCGA Cancer Genomic database (http://www.cbioportal.org), 14.7% cases displayed ERLIN2 gene alteration. Excluding 107 cases deceased, the survival rates of the cases with ERLIN2 gene alteration (*n*=127) were significantly lower than those without ERLIN2 gene alteration (*n*=720), based on overall survival Kaplan–Meier Estimate. The *P-*values were calculated by Log rank Test.

Having established the roles of ERLIN2 in stabilizing microtubules and Cyclin B1 complex, we asked whether downregulation of ERLIN2 could affect cancer cell proliferation and malignancy. Cell proliferation assay showed that ERLIN2-knockdown SUM225 breast cancer cells exhibited significantly reduced proliferation rates, compared with the control cells (Figure 7a). FACS analysis revealed that ERLIN2-knockdown SUM225 population had significantly more cells in G2/M phase and fewer in G1 phase, compared to that of the control SUM225 cells, after a 6-day culture (Figure 7b). Next, we performed a 50-day breast tumor xenograft study with nude mice to assess the impact of ERLIN2 knockdown in breast cancer malignancy *in vivo.* ERLIN2-knockdown and control SUM225 cells were orthotopically implanted into the mammary fat pads of the left and right flanks, respectively, of NOD/SCID mice. From 25 days after the implantation, the growth rates of ERLIN2-knockdown tumors were remarkably reduced compared with those of the control tumors (Figure 7c). The xenografted ERLIN2-knockdown breast tumors displayed diminished sizes at 7 weeks after the transplantation (Figure 7d). Furthermore, we evaluated the involvement of ERLIN2 in breast cancer therapy resistance by treating ERLIN2 knockdown and control SUM225 cells with the anticancer drug Bortezomib [42]. The ERLIN2-knockdown breast cancer cells were exquisitely more sensitive to low doses of Bortezomib treatment, with an IC_50_ value as low as 0.467 ng ml^−1^, compared with the control breast cancer cells with an IC_50_ of 8.027 (Figure 7e). This result suggested that ERLIN2 may have an important role in breast cancer resistance to anticancer drugs. Consistently, analysis of human primary breast cancer database (provided by TCGA Cancer Genomic, Bethesda, MD, USA) indicated that the survival rates of human breast cancer patients with the *ERLIN2* gene alteration were significantly lower than those without the *ERLIN2* gene alteration (Figure 7f), thus confirming a significant role of ERLIN2 in human breast cancer malignancy.

## Discussion

**Figure 8:**
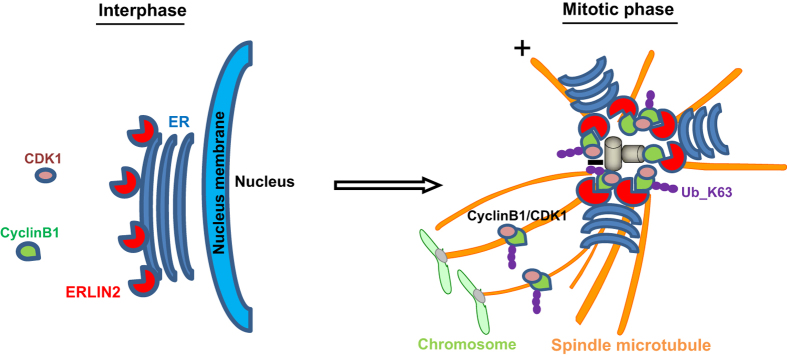
Schematic working model for the regulation of cell cycle progression by endoplasmic reticulum lipid raft-associated protein 2 (ERLIN2). During interphase, nuclear membrane is intact and Cyclin B1 levels are low. In the M phase, nuclear membrane is broken down, ER is redistributed, and spindle microtubules are formed. By simultaneously binding to spindle microtubules, Cyclin B1, and Cdk1, ERLIN2 stabilizes spindle microtubules and Cyclin B1/Cdk1 complex by promoting K63-linked ubiquitination of Cyclin B1. By stabilizing microtubules and sustaining high Cyclin B1/Cdk1 activity, ERLIN2 modulates cell cycle progression and cell growth rates.

In this study, we revealed that ERLIN2 is a developmentally regulated, ER-resident MAP that stabilizes Cyclin B1 and regulates cell cycle progression (Figure 8). Our major findings include: (1) ERLIN2 is developmentally regulated in normal tissues and overexpressed in aggressive human breast cancer tissues; (2) ERLIN2 binds to and stabilizes microtubules by interacting with α-tubulin, but not β-tubulin, through its SPFH domain; (3) ERLIN2 complexes with Cyclin B1, Cdk1 and α-tubulin in G2/M phase during the cell division cycle; (4) ERLIN2 interacts with Cyclin B1 and promotes K63-linked ubiquitination of Cyclin B1; (5) ERLIN2 stabilizes Cyclin B1 and thus sustains Cyclin B1/Cdk1 activity in G2/M phase; (6) ERLIN2 can modulate cell cycle progression; and (7) Knockdown of ERLIN2 mitigates breast cancer cell proliferation and malignancy and increases sensitivity of breast cancer cells to the anticancer drug Bortezomib. These findings represent a discovery of a novel regulatory mechanism involving an ER protein in cell cycle progression and have important implications in the understanding and treatment of aggressive human breast cancers.

ER displays dynamic redistribution during mitosis via recruitment to mitotic spindle poles from prophase to metaphase [43]. On the basis of our study, ERLIN2 is so far the first-identified ER protein that simultaneously interacts with spindle microtubules and Cyclin B1/CDK1 to regulate the cell division cycle. As an ER lipid raft-associated protein, ERLIN2 does not interact with Cyclin B1 or Cdk1 during interphase (Figure 4). As the cell cycle progresses into the M phase, ER undergoes both spatial reorganization and structural transformation and is recruited to mitotic spindle poles [44]. G2/M-specific Cyclin B complexes with Cdk1 kinase to form the M phase-promoting factor (MPF), which associates with microtubules in the mitotic spindle through interacting with MAPs [19]. Our study demonstrated that ERLIN2, as an ER-associated MAP, simultaneously interacts with α-tubulin, Cyclin B1 and Cdk1 during M phase, (Figure 4a). Importantly, through the interactions, ERLIN2 promotes K63-linked Cyclin B1 ubiquitination, thus stabilizing Cyclin B1 complex by preventing proteasome-mediated Cyclin B1 degradation [39] (Figure 5). It is known that the spindle assembly checkpoint formed at the mitotic phase inhibits ubiquitin ligase activity of anaphase-promoting complex, thereby preventing premature Cyclin B1 destruction and Cdk1 inactivation [37, 38]. On the basis of our study, ERLIN2 likely acts as a binding partner and microtubule stabilizer by interacting with α-tubulin, Cyclin B1 and Cdk1, and thus contributes to spindle assembly or maintenance (Figure 8). Indeed, this notion is supported by the published proteomics data. ERLIN2 was detected in mitotic spindle proteomes of both CHO and HeLa cells [45]. Interestingly, ERLIN2 was unique to the mitotic spindle proteome, when compared with the mid-body proteome of CHO cells [46, 47]. On the other hand, progressive loss of Cyclin B1/Cdk1 activity through proteasome-mediated degradation is essential for the mitotic phase exit and completion of cell division. Whether ERLIN2 also interferes with the ubiquitination-mediated Cyclin B1 degradation is an interesting question to be investigated in the future. It is well documented that Cyclin B1 is unregulated in cancer cells where overexpression of Cyclin B1 may contribute to uncontrolled cell proliferation [21–23]. As for the human breast cancer cells (SUM225) in which endogenous ERLIN2 is overexpressed, the cancer cells likely utilize ERLIN2 to remodel the microtubule structure and cell division cycle, which help the cancer cells gain growth advantage and drug resistance. This notion is supported by the evidence that ERLIN2 knockdown can significantly suppress cancer cell proliferation and malignancy (Figure 7). How the cancer cells proceed G2/M phase exit while maintaining high levels of Cyclin B1 for growth advantage is an unresolved interesting question.

Our work demonstrated that ERLIN2, as an ER-associated MAP, interacts with and stabilizes Cyclin B1 by promoting K63-linked ubiquitination. It was previously reported that ERLIN2 may act as a adapter protein to facilitate polyubiquitination of ER-associated substrates, such as inositol trisphosphate receptors or 3-hydroxy-3-methylglutaryl-CoA reductase [10–12]. It is conceivable that ERLIN2 may complex with ubiquitination substrates and retrotranslocates the substrates to further interact with E3 ligases and other ubiquitination related enzymes. ERLIN2 likely associates rapidly with Cyclin B1 during G2/M phase and may act as a ‘recognition factor’ that selects Cyclin B1 to be ubiquitinated at specifically exposed lysine residues. As for the involvement of K63-linked ubiquitination in protein stabilization, it was reported that cellular proteins conjugated to K63‐ubiquitin chains are protected from the proteasome-mediated degradation and directed to the endosomal–lysosomal pathway [38]. In addition, our study suggested that the interactions between ERLIN2, microtubules, and Cyclin B1 are mediated through the SPFH domain of ERLIN2 protein (Figure 3, Supplementary Figure S4 and S5). ERLIN2 proteins across mammalian species possess three conserved cyclin-binding motifs in the SPFH domains (Supplementary Figure S4). ERLIN2 is the first-identified SPFH domain-containing protein that regulates cell cycle through interacting with both microtubules and cyclin proteins. It will be intriguing to determine whether ERLIN2 can also functionally interact with the other cyclin proteins, such as Cyclin D or Cyclin E.

We previously demonstrated that ERLIN2 supports breast cancer malignancy by facilitating cancer cell adaptation to cellular stress and accumulation of cytosolic lipid droplets [13]. The dynamics and stability of microtubules is an important control factor for cytosolic lipid droplet deposition and metabolism [48]. Apparently, the association of ERLIN2 with microtubules is consistent with the role of ERLIN2 in aiding cancer cell adaptation to cellular stress and cytosolic lipid droplet storage. In addition, our study showed that ERLIN2 is a spatially and temporally regulated MAP factor (Figure 1). In mouse CNS, ERLIN2 expression reaches peak levels in mouse cerebrum, cerebellum and spinal cord at P7, but later becomes undetectable in adult animals. The developmentally regulated expression and neuron-specific distribution of ERLIN2 in CNS are consistent with the neurological clinical symptoms of human neuronal disease caused by ERLIN2 mutations [15, 25]. For the future research, it is important to elucidate the pathophysiological involvements of ERLIN2 in neuronal diseases.

## Materials and methods

### Materials

Chemicals were purchased from Sigma (St Louis, MO, USA) unless indicated otherwise. Antibodies against GAPDH and α-tubulin were purchased from Sigma. The mouse anti-Cyclin B1 and the rabbit anti-ERLIN2 antibodies were from Cell Signaling (Beverly, MA, USA). Antibodies against V5 were from Invitrogen (Grand Island, NY, USA). NeuN antibody was from EMD Millipore (Billerica, MA, USA). Acetylated-α-tubulin was from Abcam (Cambridge, MA, USA). The rabbit anti-Cyclin B1, mouse anti-HA and mouse anti-Cdk1 antibodies were purchased from Santa Cruz Biotechnology (Dallas, TX, USA). The mouse anti-Cdc27 antibody was from BD transduction Laboratary (San Jose, CA, USA). Bortezomib (B-1408) was from LC Laboratories (Woburn, MA, USA).

### Cell culture and transduction of cells

Human breast cancer cell lines SUM225 were cultured as previously described [24]. CHO-k1 cell line was cultured in Ham’s F12 media containing 10% fetal bovine serum, glutamine and antibiotics. Huh-7 cell line was cultured in Dulbecco’s modified Eagle medium containing 10% fetal bovine serum, and glutamine. All cell lines were maintained at 37 °C, in a 5% CO2 environment. The lentiviral expression construct expressing the human ERLIN2 tagged with or without V5 (pLenti-ERLIN2) was established as previously described [49]. The pLenti-ERLIN2 virus was used to infect CHO-k1 or Huh-7 cells to produce CHO or Huh-7 cell lines that stably express different levels of V5-tagged ERLIN2. The pLenti-LacZ virus was included as an experimental control. Selection with 10 μg ml^−1^ blasticidin was started from 48 h after infection.

### Microtubule co-sedimentation assay

Brain tissues from postnatal wild-type mice of 7-day-old were homogenized in cold PEM buffer with protease inhibitors and centrifuged at 4 °C for 1 h at 180 000 *g*. The supernatant was removed as crude microtubules. Membrane proteins including endogenous depolymerized tubulins subunits were extracted from Huh7 cells overexpressing ERLIN2 using Subcellular Protein Fractionation Kit for cultured cells (Thermo Scientific Waltham, MA, USA). Equal volumes of the membrane proteins were incubated at 0 °C to depolymerize endogenous microtubule or incubated at 37 °C to polymerize endogenous microtubule. In addition, equal volumes of the membrane proteins with addition of exogenous crude microtubules at a ratio of 3:1 were used to incubate with 1 mM GTP and dimethyl sulfoxide, 10 μM nocodazole or 20 μM taxol at room temperature for 30 min to achieve depolymerization or polymerization of the microtubules. The samples were then centrifuged through a cushion buffer containing 40% glycerol at 100 000 *g* for 30 min at room temperature to collect the polymerized microtubule pellet (P) and supernatant (S), which were then resuspended in gel loading buffer for western blot analysis.

### Cell cycle synchronization and flow cytometry analysis (FACS)

CHO cells were arrested at quiescent by 0.1% serum starvation for 64 h followed by stimulation with 10% fetal bovine serum for different times to allow cell cycle reentry. To obtain cells in G0, G1 and S phase, cells were allowed to reenter the cycle for 0, 6 and 10 h. Cells in G2/M phase were obtained by treating the cells with 125 ng ml^−1^ nocodazole for 16 h. Cell cycle distribution was detected by propidium iodide staining using BD LSR II Analyzer, BD Biosciences San Jose, CA, USA. FACS cell cycle analysis was done blindly using ModFitLT software version 2.0, Topsham, ME, USA. Live cell DNA content and cell cycle distribution was done using the VybrantDyeCycle Orange, Life Technologies, Grand Island, NY, USA with BD FACS Array Bioanalyzer (BD Biosciences).

### Plasmid expression vectors for Ub and mutant Ub isoforms

Plasmid vector pRK5–HA–ubiquitin (Ub) (wild-type) was purchased from Addgene (Cambridge, MA, USA). HA-tagged Ub–K48 or Ub–K63 was made by mutagenesis in which only Lysine 48 (K48) or Lysine 63 (K63) was kept and the rest Lysine (K) residues were mutated to Arginine (R). Conversely, HA-tagged Ub–K48R or Ub–K63R was generated by mutating Lysine 48 or Lysine 63 to Arginine, respectively, and remaining the rest Lysine intact.

### Cycloheximide translation inhibition analysis of Cyclin B1 protein stability

ERLIN2 overexpresing (CHO-13) and control CHO cell lines were treated with 10 μg ml^−1^ cycloheximide and collected at 0, 30, 60, 90 and 120 min after the treatment. Immediately before addition of cycloheximide, cells were treated with 120 ng ml^−1^ of nocodazole for 16 h. To confirm that the decrease in Cyclin B1 protein levels is a result of protesome-mediated protein degredation, 5 μM of MG132 (Sigma) were added together with nocodazole. The protein lysates collected from the CHO cells were subjected to western blot analysis to determine the levels of Cyclin B1 and β-actin (loading control). Protein signal intensities, determined by western blot analysis, were quantified through Quantity One software, Bio-Rad, Hercules, CA, USA. After normlization to β-actin levels, fold changes of Cyclin B1 protein levels were calculated by comparing the Cyclin B1 protein level in MG132-treated CHO control cells (defined as 1).

### Animal implantation experiment

To evaluate the effect of ERLIN2 knockdown on human breast cancer malignancy, we performed *in vivo* breast cancer cell implantation experiments using xenograft mouse models of breast cancer. For these experiments, human breast cancer cell line SUM225 with or without ERLIN2 shRNA knockdown will be collected, counted and resuspended in Matrigel solution. The cells will be orthotopically transplanted into the mammary fat pads of the left and right flanks of nude mice. Tumor growth (sizes) were monitored every 3 days. Mice were euthanized at 7 weeks after injection, and tumor volumes were determined.

### Cell viability, dose–response and IC_50_ determination

ERLIN2-knockdown and control SUM225 cells were treated with Bortezomib (PS341) at the concentrration ranging from 0.00128, 0.0064, 0.032, 0.16, 0.8, 4, 20 to 100 ng ml^−1^ (serial 5:1 dilutions). The cells were incubated with Bortezomib for 72 h. After the incubation period, MTT assay was performed to determine cell viability. Each treatment point was performed in quadruplicate and the cell survival dose–response were analyzed by the GraphPad Prism software (V6), La Jolla, CA, USA. The dose–response curves were generated through nonlinear regression based on log(dose) vs cell viability response (variable slope). IC_50_ (inhibitory concentration with 50% cell viability) values, indicating the concentration of Bortezomib that inhibited 50% of cell viability compared with untreated control, were calculated based on the dose–response curves.

For a full description of Materials and Methods used in this work, see Supplementary Information.

## References

[bib1] Yang ZQ , Streicher KL , Ray ME , Abrams J , Ethier SP . Multiple interacting oncogenes on the 8p11-p12 amplicon in human breast cancer. Cancer Res 2006; 66: 11632–11643. 17178857 10.1158/0008-5472.CAN-06-2946

[bib2] Yildirim Y , Orhan EK , Iseri SA et al. A frameshift mutation of ERLIN2 in recessive intellectual disability, motor dysfunction and multiple joint contractures. Hum Mol Genet 2011; 20: 1886–1892. 21330303 10.1093/hmg/ddr070

[bib3] Finsterer J , Loscher W , Quasthoff S , Wanschitz J , Auer-Grumbach M , Stevanin G . Hereditary spastic paraplegias with autosomal dominant, recessive, X-linked, or maternal trait of inheritance. J Neurol Sci 2012; 318: 1–18. 22554690 10.1016/j.jns.2012.03.025

[bib4] Alazami AM , Adly N , Al Dhalaan H , Alkuraya FS . A nullimorphic ERLIN2 mutation defines a complicated hereditary spastic paraplegia locus (SPG18). Neurogenetics 2011; 12: 333–336. 21796390 10.1007/s10048-011-0291-8 PMC3215864

[bib5] Yang ZQ , Liu G , Bollig-Fischer A , Giroux CN , Ethier SP . Transforming properties of 8p11-12 amplified genes in human breast cancer. Cancer Res 2010; 70: 8487–8497. 20940404 10.1158/0008-5472.CAN-10-1013 PMC3089754

[bib6] Gelsi-Boyer V , Orsetti B , Cervera N et al. Comprehensive profiling of 8p11-12 amplification in breast cancer. Mol Cancer Res 2005; 3: 655–667. 16380503 10.1158/1541-7786.MCR-05-0128

[bib7] Garcia MJ , Pole JC , Chin SF et al. A 1Mb minimal amplicon at 8p11-12 in breast cancer identifies new candidate oncogenes. Oncogene 2005; 24: 5235–5245. 15897872 10.1038/sj.onc.1208741

[bib8] Holland DG , Burleigh A , Git A et al. ZNF703 is a common Luminal B breast cancer oncogene that differentially regulates luminal and basal progenitors in human mammary epithelium. EMBO Mol Med 2011; 3: 167–180. 21337521 10.1002/emmm.201100122 PMC3395113

[bib9] Sircoulomb F , Nicolas N , Ferrari A et al. ZNF703 gene amplification at 8p12 specifies luminal B breast cancer. EMBO Mol Med 2011; 3: 153–166. 21328542 10.1002/emmm.201100121 PMC3395112

[bib10] Pearce MM , Wormer DB , Wilkens S , Wojcikiewicz RJ . An endoplasmic reticulum (ER) membrane complex composed of SPFH1 and SPFH2 mediates the ER-associated degradation of inositol 1,4,5-trisphosphate receptors. J Biol Chem 2009; 284: 10433–10445. 19240031 10.1074/jbc.M809801200 PMC2667730

[bib11] Pearce MM , Wang Y , Kelley GG , Wojcikiewicz RJ . SPFH2 mediates the endoplasmic reticulum-associated degradation of inositol 1,4,5-trisphosphate receptors and other substrates in mammalian cells. J Biol Chem 2007; 282: 20104–20115. 17502376 10.1074/jbc.M701862200

[bib12] Jo Y , Sguigna PV , DeBose-Boyd RA . Membrane-associated ubiquitin ligase complex containing gp78 mediates sterol-accelerated degradation of 3-hydroxy-3-methylglutaryl-coenzyme A reductase. J Biol Chem 2011; 286: 15022–15031. 21343306 10.1074/jbc.M110.211326 PMC3083207

[bib13] Wang G , Zhang X , Lee JS , Wang X , Yang ZQ , Zhang K . Endoplasmic reticulum factor ERLIN2 regulates cytosolic lipid content in cancer cells. Biochem J 2012; 446: 415–425. 22690709 10.1042/BJ20112050 PMC3806481

[bib14] Huber MD , Vesely PW , Datta K , Gerace L . Erlins restrict SREBP activation in the ER and regulate cellular cholesterol homeostasis. J Cell Biol 2013; 203: 427–436. 24217618 10.1083/jcb.201305076 PMC3824017

[bib15] Al-Saif A , Bohlega S , Al-Mohanna F . Loss of ERLIN2 function leads to juvenile primary lateral sclerosis. Ann Neurol 2012; 72: 510–516. 23109145 10.1002/ana.23641

[bib16] Belzil VV , Rouleau GA . Endoplasmic reticulum lipid rafts and upper motor neuron degeneration. Ann Neurol 2012; 72: 479–480. 23109142 10.1002/ana.23678

[bib17] Avila J , Dominguez J , Diaz-Nido J . Regulation of microtubule dynamics by microtubule-associated protein expression and phosphorylation during neuronal development. Int J Dev Biol 1994; 38: 13–25. 8074993

[bib18] Bhat KM , Setaluri V . Microtubule-associated proteins as targets in cancer chemotherapy. Clin Cancer Res 2007; 13: 2849–2854. 17504982 10.1158/1078-0432.CCR-06-3040

[bib19] Ookata K , Hisanaga S , Bulinski JC et al. Cyclin B interaction with microtubule-associated protein 4 (MAP4) targets p34cdc2 kinase to microtubules and is a potential regulator of M-phase microtubule dynamics. J Cell Biol 1995; 128: 849–862. 7876309 10.1083/jcb.128.5.849 PMC2120387

[bib20] Ookata K , Hisanaga S , Okano T , Tachibana K , Kishimoto T . Relocation and distinct subcellular localization of p34cdc2-cyclin B complex at meiosis reinitiation in starfish oocytes. EMBO J 1992; 11: 1763–1772. 1316272 10.1002/j.1460-2075.1992.tb05228.x PMC556634

[bib21] Suzuki T , Urano T , Miki Y et al. Nuclear cyclin B1 in human breast carcinoma as a potent prognostic factor. Cancer Sci 2007; 98: 644–651. 17359284 10.1111/j.1349-7006.2007.00444.x PMC11159733

[bib22] Kawamoto H , Koizumi H , Uchikoshi T . Expression of the G2-M checkpoint regulators cyclin B1 and cdc2 in nonmalignant and malignant human breast lesions: immunocytochemical and quantitative image analyses. Am J Pathol 1997; 150: 15–23. 9006317 PMC1858517

[bib23] Dutta A , Chandra R , Leiter LM , Lester S . Cyclins as markers of tumor proliferation: immunocytochemical studies in breast cancer. Proc Natl Acad Sci USA 1995; 92: 5386–5390. 7539916 10.1073/pnas.92.12.5386 PMC41699

[bib24] Wang G , Liu G , Wang X et al. ERLIN2 promotes breast cancer cell survival by modulating endoplasmic reticulum stress pathways. BMC Cancer 2012; 12: 225. 22681620 10.1186/1471-2407-12-225 PMC3732090

[bib25] Wakil SM , Bohlega S , Hagos S et al. A novel splice site mutation in ERLIN2 causes hereditary spastic paraplegia in a Saudi family. Eur J Med Genet 2013; 56: 43–45. 23085305 10.1016/j.ejmg.2012.10.003

[bib26] Sakakibara A , Ando R , Sapir T , Tanaka T . Microtubule dynamics in neuronal morphogenesis. Open Biol 2013; 3: 130061. 23864552 10.1098/rsob.130061 PMC3728923

[bib27] Downing KH , Nogales E . Tubulin and microtubule structure. Curr Opin Cell Biol 1998; 10: 16–22. 9484591 10.1016/s0955-0674(98)80082-3

[bib28] Lefevre J , Savarin P , Gans P et al. Structural basis for the association of MAP6 protein with microtubules and its regulation by calmodulin. J Biol Chem 2013; 288: 24910–24922. 23831686 10.1074/jbc.M113.457267 PMC3750186

[bib29] Poruchynsky MS , Sackett DL , Robey RW , Ward Y , Annunziata C , Fojo T . Proteasome inhibitors increase tubulin polymerization and stabilization in tissue culture cells: a possible mechanism contributing to peripheral neuropathy and cellular toxicity following proteasome inhibition. Cell Cycle 2008; 7: 940–949. 18414063 10.4161/cc.7.7.5625 PMC9416319

[bib30] Song Y , Kirkpatrick LL , Schilling AB et al. Transglutaminase and polyamination of tubulin: posttranslational modification for stabilizing axonal microtubules. Neuron 2013; 78: 109–123. 23583110 10.1016/j.neuron.2013.01.036 PMC3627183

[bib31] Cassimeris LU , Wadsworth P , Salmon ED . Dynamics of microtubule depolymerization in monocytes. J Cell Biol 1986; 102: 2023–2032. 3519619 10.1083/jcb.102.6.2023 PMC2114271

[bib32] Shim SY , Wang J , Asada N et al. Protein 600 is a microtubule/endoplasmic reticulum-associated protein in CNS neurons. J Neurosci 2008; 28: 3604–3614. 18385319 10.1523/JNEUROSCI.5278-07.2008 PMC6671073

[bib33] Yuan J , Yan R , Kramer A et al. Cyclin B1 depletion inhibits proliferation and induces apoptosis in human tumor cells. Oncogene 2004; 23: 5843–5852. 15208674 10.1038/sj.onc.1207757

[bib34] Ookata K , Hisanaga S , Okumura E , Kishimoto T . Association of p34cdc2/cyclin B complex with microtubules in starfish oocytes. J Cell Sci 1993; 105 (Pt 4): 873–881. 8227209 10.1242/jcs.105.4.873

[bib35] Gawlinski P , Nikolay R , Goursot C et al. The *Drosophila* mitotic inhibitor Fruhstart specifically binds to the hydrophobic patch of cyclins. EMBO Rep 2007; 8: 490–496. 17431409 10.1038/sj.embor.7400948 PMC1866202

[bib36] Lindqvist A , van Zon W , Karlsson Rosenthal C , Wolthuis RM . Cyclin B1-Cdk1 activation continues after centrosome separation to control mitotic progression. PLoS Biol 2007; 5: e123. 17472438 10.1371/journal.pbio.0050123 PMC1858714

[bib37] Pines J . Mitosis: a matter of getting rid of the right protein at the right time. Trends Cell Biol 2006; 16: 55–63. 16337124 10.1016/j.tcb.2005.11.006

[bib38] Taylor SS , Scott MI , Holland AJ . The spindle checkpoint: a quality control mechanism which ensures accurate chromosome segregation. Chromosome Res 2004; 12: 599–616. 15289666 10.1023/B:CHRO.0000036610.78380.51

[bib39] Nathan JA , Kim HT , Ting L , Gygi SP , Goldberg AL . Why do cellular proteins linked to K63-polyubiquitin chains not associate with proteasomes? EMBO J 2013; 32: 552–565. 23314748 10.1038/emboj.2012.354 PMC3579138

[bib40] Deng L , Wang C , Spencer E et al. Activation of the IkappaB kinase complex by TRAF6 requires a dimeric ubiquitin-conjugating enzyme complex and a unique polyubiquitin chain. Cell 2000; 103: 351–361. 11057907 10.1016/s0092-8674(00)00126-4

[bib41] Patra D , Dunphy WG . Xe-p9, a Xenopus Suc1/Cks protein, is essential for the Cdc2-dependent phosphorylation of the anaphase- promoting complex at mitosis. Genes Dev 1998; 12: 2549–2559. 9716407 10.1101/gad.12.16.2549 PMC317096

[bib42] Feng R , Li S , Lu C et al. Targeting the microtubular network as a new antimyeloma strategy. Mol Cancer Ther 2011; 10: 1886–1896. 21825007 10.1158/1535-7163.MCT-11-0234

[bib43] Curran MP , McKeage K . Bortezomib: a review of its use in patients with multiple myeloma. Drugs 2009; 69: 859–888. 19441872 10.2165/00003495-200969070-00006

[bib44] Bobinnec Y , Marcaillou C , Morin X , Debec A . Dynamics of the endoplasmic reticulum during early development of Drosophila melanogaster. Cell Motil Cytoskeleton 2003; 54: 217–225. 12589680 10.1002/cm.10094

[bib45] Puhka M , Joensuu M , Vihinen H , Belevich I , Jokitalo E . Progressive sheet-to-tubule transformation is a general mechanism for endoplasmic reticulum partitioning in dividing mammalian cells. Mol Biol Cell 2012; 23: 2424–2432. 22573885 10.1091/mbc.E10-12-0950 PMC3386207

[bib46] Sauer G , Korner R , Hanisch A , Ries A , Nigg EA , Sillje HH . Proteome analysis of the human mitotic spindle. Mol Cell Proteomics 2005; 4: 35–43. 15561729 10.1074/mcp.M400158-MCP200

[bib47] Bonner MK , Poole DS , Xu T , Sarkeshik A , Yates JR 3rd , Skop AR . Mitotic spindle proteomics in Chinese hamster ovary cells. PLoS One 2011; 6: e20489. 21647379 10.1371/journal.pone.0020489 PMC3103581

[bib48] Skop AR , Liu H , Yates J 3rd , Meyer BJ , Heald R . Dissection of the mammalian midbody proteome reveals conserved cytokinesis mechanisms. Science 2004; 305: 61–66. 15166316 10.1126/science.1097931 PMC3679889

[bib49] Zhang X , Zhang K . Endoplasmic Reticulum Stress-Associated Lipid Droplet Formation and Type II Diabetes. Biochem Res Int 2012; 2012: 247275. 22506114 10.1155/2012/247275 PMC3299243

