# Supplementary material for: A novel ER–microtubule-binding protein, ERLIN2, stabilizes Cyclin B1 and regulates cell cycle progression
Source: Cell Discov. 2015 Sep 8;1:15024–. doi: 10.1038/celldisc.2015.24 (PMC4860859; doi:10.1038/celldisc.2015.24)
Supplement: Supplementary Information [file celldisc201524-s1.pdf]

Supplemental Information

"A Novel ER-Microtubule Binding Protein, ERLIN2, Stabilizes Cyclin B1 and Regulates Cell Cycle Progression" by Zhang et al.

Material and Methods

Plasmid construction - The cDNAs expressing ERLIN2 and its truncation mutants were subcloned into the pCR2.1 TOPO vector (Invitrogen). To express green fluorescent protein (GFP)-fused ERLIN2 proteins, ERLIN2 and its deletion mutants were subcloned into the pEGFP-N1 expression vector (Clontech). Cyclin B1and its deletion mutants tagged with flag were provided by Dr. Deyu Fang. All constructs were verified by sequencing.

Lentivirus-mediated shRNA knockdown - Expression of the human *ERLIN2* gene in SUM225 or Huh-7 was knocked down using the Expression Arrest GIPZ lentiviral shRNAmir system (OpenBiosystems, Huntsville, AL) as previously described (Wang et al., 2012). For cell infection, viral supernatants were supplemented with 6 μg/mL polybrene and incubated with cells for 24 hours. Cells expressing shRNA were cultured under puromycin for 2-3 weeks to select knockdown stable cell lines prior to functional studies (cell proliferation assays).

Cell culture and transduction of cells - Human breast cancer cell lines SUM225 were cultured as previously described (Wang et al., 2012). CHO-k1 cell line was cultured in Ham's F12 media containing 10% fetal bovine serum, glutamine, and antibiotics. Huh-7 cell line was cultured in Dulbecco's modified Eagle medium (DMEM) containing 10% fetal bovine serum, and glutamine.

All cell lines were maintained at 37°C, in a 5% CO2 environment. The lentiviral expression construct expressing the human ERLIN2 tagged with or without V5 (pLenti-ERLIN2) was established as previously described (Wang et al., 2012). The pLenti-ERLIN2 virus was used to infect CHO-k1 or Huh-7 cells to produce CHO or Huh-7 cell lines that stably express different levels of V5 tagged-ERLIN2. The pLenti-LacZ virus was included as an experimental control. Selection with 10 μg/mL blasticidin was started from 48 hours after infection.

Western Blot and Immunoprecipitation (IP)-Western blot analyses - To determine expression levels of ERLIN2, α-tubulin, Cyclin B1, or GAPDH, total cell lysates were prepared from cultured cells or various tissues from wild-type mice using NP-40 lysis buffer. Denatured proteins were separated by SDS-PAGE on 10% Tris-glycine polyacrylamide gels and transferred onto a 0.45-mm PVDF membrane (Fisher Scientific). Membrane-bound antibodies were detected by an enhanced chemiluminescence detection reagent (Fisher Scientific). For IP-Western blot analysis, total protein lysates from in vitro cultured cells were immunoprecipitated with anti-α-tubulin, anti-β-tubulin, anti-Cyclin B1, anti-CDK1 or anti-ERLIN2 antibodies, followed by Western blot analysis using the anti-ERLIN2 or anti-α-tubulin antibody to detect protein interactions. Data shown was a representative of at least 3 independent experiments.

Immunofluorescence - Indirect immunofluorescence was performed as described previously (Zhang et al., 2008). In brief, cells were grown on glass coverslips, fixed with 4% paraformaldehyde in PBS for 10min followed by blocking with 5% normal goat serum in 0.1 Triton X-100/PBS before incubation with primary antibodies. Secondary antibodies used were Alexa Fluor 594- or Fluor 488-conjugated chicken anti-mouse or anti-rabbit IgG (Invitrogen).

Slides were mounted with SlowFade Gold anti-fade reagent containing DAPI. Fluorescent signals were observed and photographed using a fluorescence microscope, and the images were acquired with AxioVisionrel 4.6.

Tissue array and immunohistochemistry (IHC) staining - Human breast cancer tissue array was obtained from Nuclea Biotechnologies (US Biolab, Washington). Immunohistochemistry was performed on tumor tissue sections using the standard laboratory protocols. Briefly, after deparaffinizing and hydrating with phosphate-buffered saline (PBS) buffer (pH 7.4), the sections were pretreated with hydrogen peroxide (3%) for 10 min to remove endogenous peroxidase, followed by antigen retrieval via steam bath for 20 min in EDTA. The rabbit anti-ERLIN2 primary antibody was applied, followed by washing and incubation with the biotinylated secondary antibody for 30 min at room temperature. Detection was performed with diaminobenzidine (DAB) and counterstaining with Mayer hematoxylin followed by dehydration and mounting. Immunostained slides were blindly evaluated under a transmission light microscope. Areas of highest staining density were identified for evaluating the expression in tumors. ERLIN2 staining were scored on a scale of 1-3: ERLIN2 staining in <50 % of cells = 1+, intermediate staining in >50 % of cells = 2+, and strong staining in >50 % of cells = 3+.

Cell proliferation assay - Cell proliferation rates were determined using CellTiter 96 non-radioactive cell proliferation MTT assay kits (Promega). Briefly, approximately 5000 cells per well were seeded in triplicate in 96-well culture plates. After cell culture for 24, 48, and 72 hrs, 20 μl of MTT (5 mg/ml) solution was added to 200 μl of medium in each well. Cells were cultured for an additional 4 hrs to allow MTT to be well metabolized. After that, the medium was aspirated,

and 200 μl of DMSO was added into the well to dissolve the purple formazan crystals. The absorbance of the plate was measured at 570 nm using a plate reader.

Reference:

Wang, G., G. Liu, X. Wang, S. Sethi, R. Ali-Fehmi, J. Abrams, Z. Zheng, K. Zhang, S. Ethier, and Z.Q. Yang. 2012. ERLIN2 promotes breast cancer cell survival by modulating endoplasmic reticulum stress pathways. *BMC Cancer*. 12:225.

Zhang, X., C.Y. Chow, Z. Sahenk, M.E. Shy, M.H. Meisler, and J. Li. 2008. Mutation of FIG4 causes a rapidly progressive, asymmetric neuronal degeneration. *Brain*. 131:1990-2001.

Supplemental Figure legends

S-Fig 1: Analysis of the ERLIN2 gene expression in different human developmental stage based on microarray data from public domain database. Expression profiles of the ERLIN2 genes in human tissues were extracted from the European Bioinformatics Institute of European Molecular Biology Laboratory (EMBL-EBI, http://www.ebi.ac.uk/). Fold changes of expression levels of the ERLIN2 gene in human tissues were determined by normalizing to expression levels of the genes in cerebellum (which were defined as 1).

S-Fig 2: (**A**) Levels of ERLIN2 and α-tubulin in ERLIN2-knockdown (EN2 KD) and non-silence control (Nonsil) SUM225 cells, determined by Western blot analysis. (**B**) Levels of ERLIN2 and GAPDH in ERLIN2-over-expressing (EN2 OE) and control CHO cells, determined by Western blot analysis.

S-Fig 3: Predicted topology of the human ERLIN2 based on the Mobyle topology prediction program for membrane proteins (TopPred 1.10; http://mobyle.pasteur.fr). In two speculative

models, N-terminal (NH3) is located in the cytosol and the ER lumen, respectively. There are two candidate membrane-spanning (transmembrane) segments: (1) amino acids 4 to 24; and (2) amino acids 38 to 58. In both models, partial of the SPFH domain is exposed to the cytosol.

S-Fig 4: Alignment of ERLIN2 protein sequences of human, mouse, and rat species. SPFH domain is highlighted in yellow; SPFH domain sequence is underlined. KXL cyclin binding motif is highlighted in red rectangle.

S-Fig 5: (**A**) Schematic representation of different domains on ERLIN2 protein. SPFH, SPFH domain (22-226aa); oligo, oligomerization domain (228-300aa); hydro, hydrophobic patch (301-306aa). Plasmid vectors were constructed to express ERLIN2 or its deletion mutations (n306, n300, n226, n24) fused with a C-terminal GFP. (**B**) Western blot and IP-Western blot analyses of the interaction between GFP-tagged ERLIN2 or its truncated isoforms and Cyclin B1 in CHO cells. Empty vector (pEGFP-N1 vector) was included as the control (Ve-GFP). Total cell lysates from transfected CHO cells were immunoprecipated with the anti-Cyclin B1 antibody. The pull-down proteins were subjected to immunoblotting analysis using anti-GFP antibody. The levels of GFP-tagged ERLIN2 variants in total cell lysates were determined by Western blot analysis using the anti-GFP antibody (left panel). H-chain, IgG heavy chain.

S-Fig 6: Western blot analysis of phosphorylated CDC27 protein levels in ERLIN2-expressing CHO cells. Expression vectors for flag-tagged Cyclin B1 and HA-tagged Ub or its mutant isoforms carrying a single lysine residue (K48 or K63), or HA-tagged Ub isoforms with a single mutation at K48 (K48R) or at K63 (K63R), were co-transfected into the CHO cells stably expressing

exogenous ERLIN2. The CHO cells were then treated with the vehicle DMSO or Nocodazole to synchronize the cells in the G2/M phase. Western blot analyses with the total cellular protein lysates were performed to determine the levels of phosphorylated CDC27 and GAPDH.

S-Fig 7: Percentages of CHO-17 and CHO-13 cells in G0/G1, S, and G2/M phases after nocodazole treatment followed by nocodazole removal. CHO-17 and CHO-13 cells were treated with nocodazole for 16 hrs to arrest most cells in pro-metaphase by depolymerizing spindle microtubules. The culture medium containing nocodazole was then washed out and replaced with normal culture medium to allow recovery of functional spindle microtubules for 2, 5, and 8 hrs. FACS analysis was conducted with the cells collected at different time points to profile percentages of the cells in G0/G1, S, and G2/M phases upon recovery from nocodazole treatment. CV: coefficient of variation.

Zhang_S-Fig 1

Levels of the ERLIN2 mRNA in human tissues under different developmental stage

Developmental stage	ERLIN2		
	PubMed ID	T-	P-Value
Embryo at two cell stage		2.9	0.048
Embryo at eight cell stage	20219939	3.8	1.20E-02
Blastocyst		6.9	8.69E-05
Midgestation		2.9	4.00E-02
Term	17170095	-2.9	4.00E-02
Young cells		5.5	9.00E-03
Senescence	E-MEXP-2283	-5.5	9.00E-03
Juvenile		5.6	8.05E-06
Adult	E-TABM-1214	-5.6	8.05E-06
Pediatric		5.2	1.42E-04
Geriatric	15962335	-5.2	1.42E-04
Infant		3.2	2.70E-02
Adult	16089502	-3.2	2.70E-02

Zhang_S-Fig 2

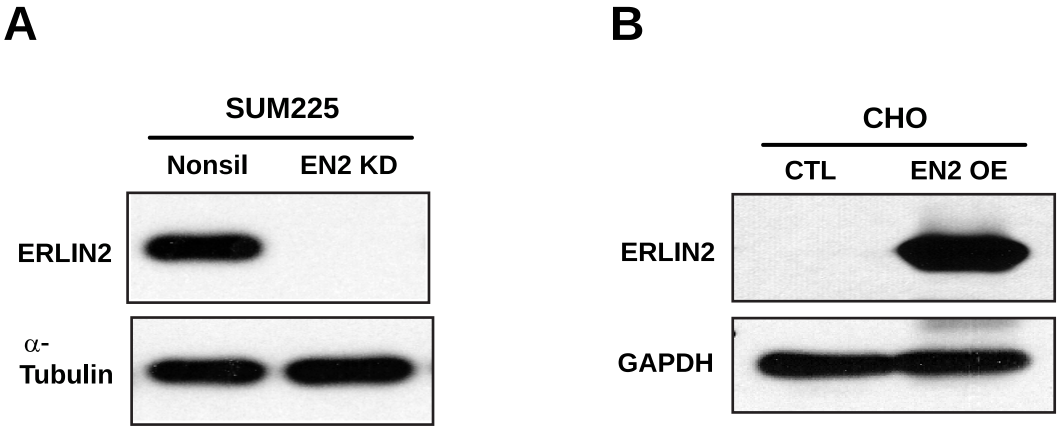
A
SUM225
Nonsil
EN2 KD
ERLIN2
α-
Tubulin
B
CHO
CTL
EN2 OE
ERLIN2
GAPDH

Zhang_S-Fig 3

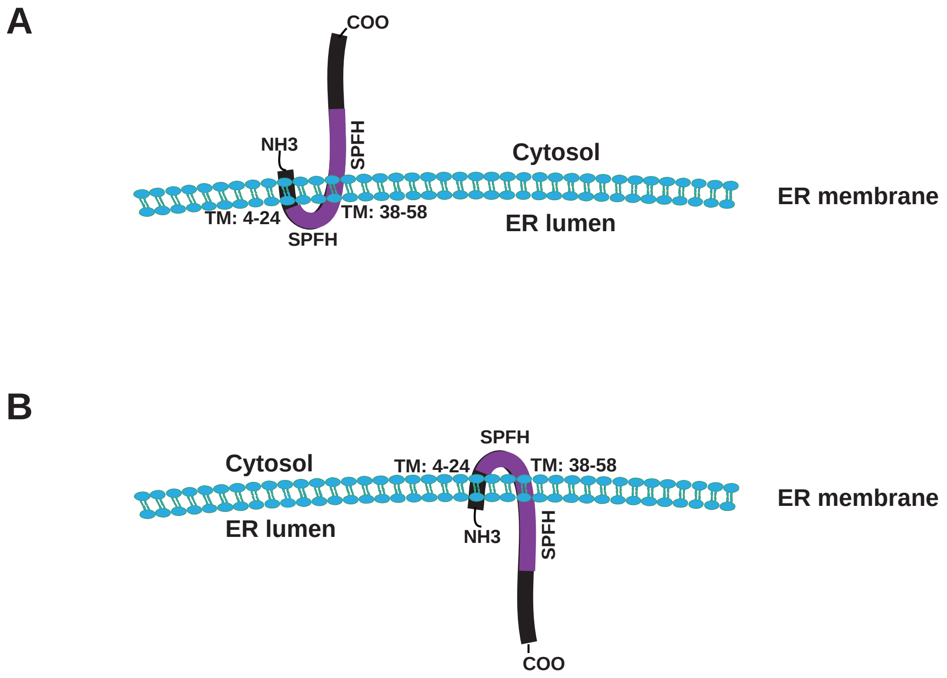
A
COO
NH3
SPFH
Cytosol
ER membrane
TM: 4-24
TM: 38-58
ER lumen
SPFH
B
SPFH
Cytosol
TM: 4-24
TM: 38-58
ER membrane
ER lumen
NH3
SPFH
COO

Alignment of the ERLIN2 protein sequences of human, mouse, and rat species

Human	1	MAQLGAVVAVASSFFCASLFSAVHKIEEGHIGVYYRGGALLTSTSGPGFHLMLPFITSYKSVQTTLQTDEVKNVPCGTSG	80
Mouse	1	MAQLGAVVAVASSFFCASLFSAVHKIEEGHIGVYYRGGALLTSTSGPGFHLMLPFITSYKSVQTTLQTDEVKNVPCGTSG	80
Rat	1	MAQLGAVVAVASSFFCASLFSAVHKIEEGHIGVYYRGGALLTSTSGPGFHLMLPFITSYKSVQTTLQTDEVKNVPCGTSG	80
Human	81	GVMIYFDRIEVVNFLVPNAVYDIVKNYTADYDKALIFNKIHHELNQFCSVHTLQEVYIELFDQIDENLKLALQQDLTSMA	160
Mouse	81	GVMIYFDRIEVVNFLVPNAVYDIVKNYTADYDKALIFNKIHHELNQFCSVHTLQEVYIELFDQIDENLKLALQQDLTSMA	160
Rat	81	GVMIYFDRIEVVNFLVPHAVYDIVKNYTADYDKALIFNKIHHELNQFCSVHTLQEVYIELFDQIDENLKLALQQDLTSMA	160
Human	161	PGLVIQAVRVTKPNIPEAIRRNYELMESEKTKLLIAAQKQKVVEKEAETERKKALIEAEKVAQVAEITYGQKVMEKETEK	240
Mouse	161	PGLVIQAVRVTKPNIPEAIRRNYELMESEKTKLLIAAQKQKVVEKEAETERKKALIEAEKVAQVAEITYGQKVMEKETEK	240
Rat	161	PGLVIQAVRVTKPNIPEAIRRNYELMESEKTKLLIAAQKQKVVEKEAETERKKALIEAEKVAQVAEITYGQKVMEKETEK	240
Human	241	KISEIEDAAFLAREKAKADAECYTAMKIAEANKLKLTPEYLQLMKYKAIASNSKIYFGKDIPNMFMDSAGSVSKQFEGLA	320
Mouse	241	KISEIEDAAFLAREKAKADAECYTALKIAEANKLKLTPEYLQLMKYKAIASNSKIYFGKDIPNMFMDSAGGLGKQFEGLS	320
Rat	241	KISEIEDAAFLAREKAKADAECYTALKIAEANKLKLTPEYLQLMKYKAIASNSKIYFGKDIPNMFMDSAGGLGKQSEGLS	320
Human	321	-DKLSFGLEDEPLETATKEN	339
Mouse	321	DDKLGFGLEDEPLEAPTKEN	340
Rat	321	-DKLGFGLEDEPLETATKDN	339

The highlighted (yellow) sequences are SPFH domains; the amino acid sites within the red rectangles are KXL cyclin-binding motifs

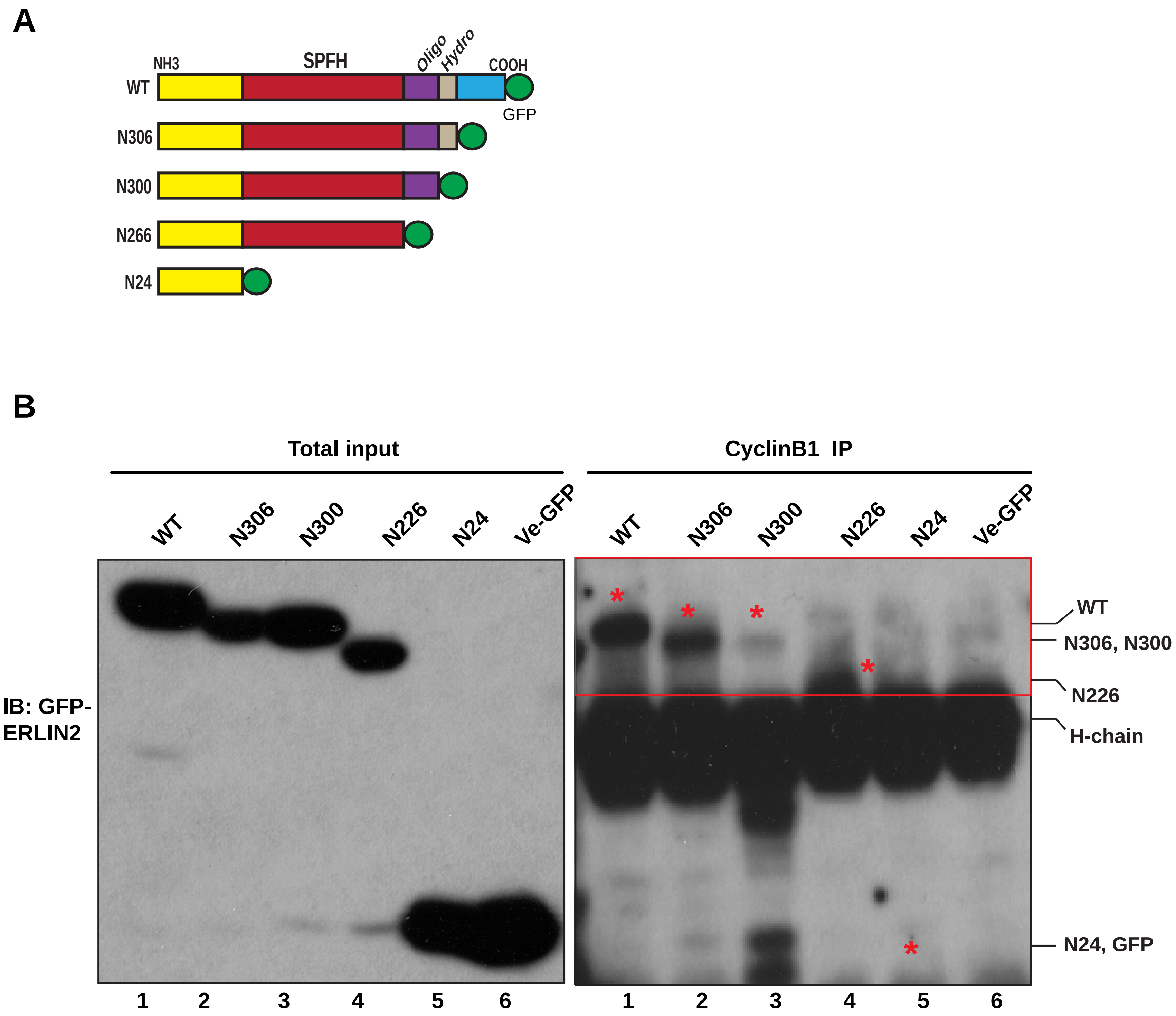
A
NH3
SPFH
Oligo
Hydro
COOH
WT
GFP
N306
N300
N266
N24
B
Total input
CyclinB1 IP
WT
N306
N300
N226
N24
Ve-GFP
IB: GFP-ERLIN2
WT
N306, N300
N226
H-chain
N24, GFP
1
2
3
4
5
6

Zhang_S-Fig 6

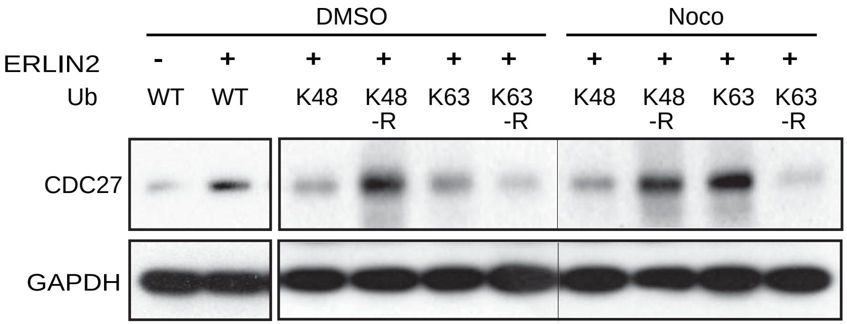
DMSO
Noco
ERLIN2
- + + + + + + + + +
Ub
WT WT K48 K48-R K63 K63-R K48 K48-R K63 K63-R
CDC27
GAPDH

Percentages of cell popultions at the G0/G1, G2/M, and S phases upon the synchronization by nocodazole treatment and at the different time points after nocodazole removal

Cell line	G0/G1 (%)	G2/M(%)	S (%)	Events	CV (%)
CHO-17 unsyn	39.06	19.2	41.47	18697	4.79
CHO-17 2hr	11.77	85.6	2.63	15606	6.98
CHO-17 5hr	18.75	81.25	0	17622	13.06
CHO-17 8hr	27.52	0	72.48	19832	9.54
CHO-13 unsyn	44.18	13.58	42.23	18826	6.68
CHO-13 2hr	44.96	50.33	4.71	17018	6.54
CHO-13 5hr	45.69	46.24	8.07	16505	9.82
CHO-13 8hr	45.38	28.7	25.92	18666	11.52
